# An effectiveness-implementation trial protocol to evaluate PrEP initiation among U.S. cisgender women using eHealth tools vs. standard care

**DOI:** 10.3389/frph.2023.1196392

**Published:** 2023-06-08

**Authors:** Lillee H. Izadi, Okeoma Mmeje, Emmanuel F. Drabo, Jamie Perin, Stephen Martin, Jenell S. Coleman

**Affiliations:** ^1^ Department of Gynecology and Obstetrics, Johns Hopkins School of Medicine, Baltimore, MD, United States; ^2^ Department of Obstetrics and Gynecology, University of Michigan Medical School, Ann Harbor, MI, United States; ^3^ Department of Health Policy and Management, Johns Hopkins Bloomberg School of Public Health, Baltimore, MD, United States; ^4^ Department of International Health, Johns Hopkins Bloomberg School of Public Health, Baltimore, MD, United States; ^5^ Department of Obstetrics and Genecology, Johns Hopkins Community Physicians, Baltimore, MD, United States.

**Keywords:** pre-exposure prophylaxis (PrEP), HIV prevention, ending the HIV epidemic (EHE), cisgender women, EHR intervention, digital health

## Abstract

**Background:**

The United States' (U.S.) initiative to End the HIV Epidemic aims to reduce new HIV infections in areas of high HIV prevalence. Despite national efforts to reduce HIV incidence, cisgender women continue to represent approximately one out of every five new HIV diagnoses in the U.S. Taking pre-exposure prophylaxis (PrEP) is an effective HIV prevention strategy; however, PrEP initiation among cisgender women is suboptimal, with only 10% of eligible women receiving PrEP prescriptions in 2019.

**Methods:**

We designed a trial to test the effectiveness of interventions to increase PrEP initiation, while evaluating the implementation strategy (hybrid type II trial) in seven obstetrics and gynecology (OB/GYN) clinics (two federally qualified health centers, three community-based, and two academic) in Baltimore, Maryland. A total of 42 OB/GYN providers will be enrolled and randomized (1:1:1) into one of three clinical trial arms (standard of care, patient-level intervention, or multi-level intervention). Eligible patients of enrolled providers will receive a sexual health questionnaire before their appointment through the electronic health record’s (EHR) patient portal. The questionnaire will be scored in three tiers (low, moderate, and high) to assess HIV risk. Patients at low risk will be offered an HIV test only, while those who score medium or high risk will be included in the clinical trial and assigned to the clinical trial arm associated with their provider. Differences in PrEP initiation, our primary outcome, across the three arms will be analyzed using generalized linear mixed-effect models with logistic regression. We will adjust results for demographic differences observed between arms and examine PrEP initiation stratified by patient’s and provider’s race and ethnicity.Additionally, a comprehensive economic analysis for each intervention will be conducted.

**Discussion:**

We hypothesize that gathering information on sensitive sexual behaviors electronically, communicating HIV risk in an understandable and relatable format to patients and OB/GYN providers, and deploying EHR alerts will increase PrEP initiation and HIV testing.

**Trial Registration:**

The trial is registered with ClinicalTrials.gov (NCT05412433) on 09 June 2022. https://clinicaltrials.gov/ct2/show/NCT05412433?term=NCT05412433&draw=2&rank=1.

## Introduction

Cisgender women comprise approximately one out of every five new HIV diagnoses in the United States in 2019, and eighty-five percent of cisgender women diagnosed with HIV attribute it to heterosexual contact (1). Consistent condom use and daily oral tenofovir disoproxil fumarate 300mg-emtricitabine 200 mg (TDF-FTC) as HIV pre-exposure prophylaxis (PrEP) are evidence-based behavioral and biomedical interventions for women to reduce their risk of HIV acquisition. Yet, cisgender women experience many barriers to accessing PrEP (2).

TDF-FTC, the only oral Food and Drug Administration (FDA)-approved PrEP medication for women, has been authorized for use since 2012 (3). However, recent data show that only up to 44% of women have heard of PrEP (2), and 1%–6% of PrEP users are women (4, 5). Underestimation of HIV risk is a leading cause of poor PrEP initiation (2). In one study, 85% of obstetrics and gynecology (OB/GYN) clinic patients in a high HIV prevalence city considered themselves low risk for HIV acquisition (2). Thus, only 41% reported using condoms when having sex with one or multiple partners in the preceding three months (2). The discordance between HIV risk perception and actual HIV risk is exacerbated when women are unaware of their male partners' HIV serostatus and HIV risk factors (2, 6). Twice as many Black men living with HIV reported having sex with both men and women compared to White men living with HIV (34% vs. 13%) (1). Provider-level reasons contributing to poor PrEP initiation among cisgender women include clinical time constraints, the inability to assess or discomfort with assessing their patients' risk for HIV acquisition, and lack of knowledge (7–9). The inability to assess HIV risk was evident in our prior data from a pregnant population in a city with high HIV prevalence. We showed that repeat HIV testing was rarely ordered (<30%) at three months, despite a state mandate (10), patients diagnosed with recurrent sexually transmitted infections (STIs), or patient report of new sexual partners and inconsistent condom use (11).

The Centers for Disease Control and Prevention's (CDC) recommendation to inform all sexually active adults and adolescents about PrEP may help to increase PrEP discussions (12), However, strategies are needed to prompt these discussions. Therefore, we designed a trial to address known patient- and provider-level barriers to PrEP, including short PrEP educational animations, electronic health record (EHR) decision support tools, and an HIV risk assessment. To ensure the interventions' effectiveness and feasibility, it is crucial to conduct a comprehensive cost analysis. This analysis will allow researchers and policymakers to evaluate an economic evaluation of the interventions compared to standard care, providing valuable insight into the allocation of limited resources in healthcare systems. By quantifying the costs associated with the intervention, including implementation, maintenance, and training expenses, stakeholders can make informed decisions about the scalability and sustainability of the proposed approach. Furthermore, a detailed cost analysis will facilitate the identification of potential barriers to adoption and inform the development of strategies to overcome these obstacles, ensuring that the intervention is both financially viable and accessible to the target population.

We hypothesize that by gathering information on sensitive sexual behaviors electronically, communicating HIV risk in an understandable and relatable format to patients and OB/GYN providers, and automating parts of clinical care (e.g., EHR alerts, facilitating HIV test ordering), PrEP initiation and HIV testing will increase.

## Methods

### Study design & setting

We designed a trial to test the effectiveness of the intervention, while evaluating the implementation strategy (hybrid type II effectiveness-implementation trial) that will be launched in seven OB/GYN clinics (three community-based, two academic, and two federally qualified health centers) affiliated with a large health system in Baltimore, Maryland. Baltimore Metropolitan Statistical Area is one of the 57 jurisdictions targeted in the Ending the HIV Epidemic (EHE) in the U.S (5). and in 2019 had an HIV incidence of 17 per 100,000 and a prevalence of 788 per 100,000 (13). Women made up 28% of new HIV diagnoses with 70% of cases transmitted via heterosexual contact and 15% via injection drug use (13).

### Study population

Inclusion criteria for patients of enrolled OB/GYN providers include age 15–65 years, scheduled for an annual gynecology exam (i.e., routine checkup), STI testing, or contraceptive counseling, and does not have an HIV diagnosis in the medical record. The age range was selected based on the U.S. Preventive Services Task Force (USPSTF) HIV Testing Recommendations and the PrEP FDA-approved age of 15 years (3, 12, 14). Women who have initiated prenatal care or are taking PrEP will be excluded.

### Study intervention

**Figure 1:**
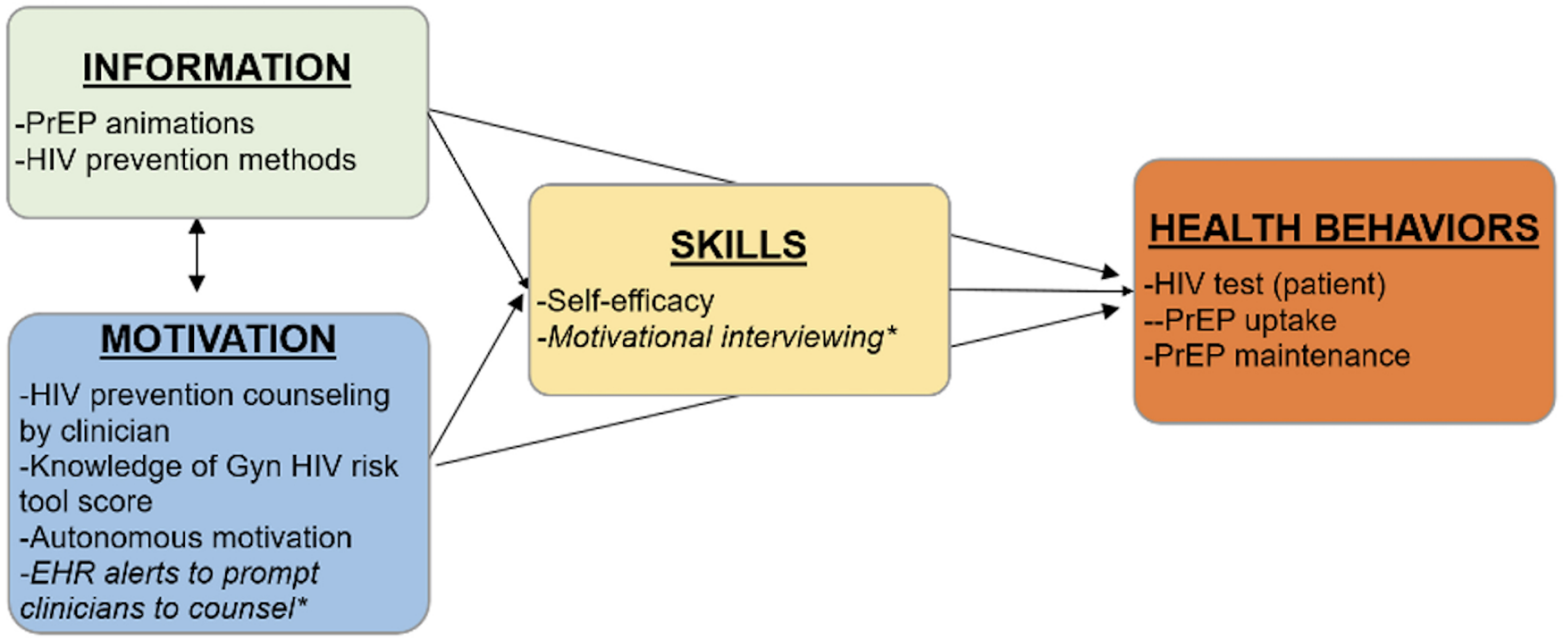
Information motivation behavioral model.

Our intervention and implementation strategies are based on the Information, Motivation, and Behavior Model (IMB) of behavior change (Figure 1). Enrolled OB/GYN providers (e.g., physicians, nurse practitioners, nurse midwives, and physician assistants) from the preselected clinics will undergo brief, 30–60-minute motivational interviewing training sessions quarterly throughout the trial. Additionally, microlearning PrEP sessions will be held to aid providers in assessing PrEP eligibility and PrEP medication management. Then, providers will be randomized to one of three arms: (1) standard-of-care arm, (2) a patient-level intervention arm, or (3) a patient-and-provider level intervention arm.

All eligible patients scheduled to receive care from an enrolled provider will be sent a link to complete an electronic sexual history questionnaire to fill out prior to their appointment. This questionnaire has 17 detailed sexual history questions [e.g., type of sex (anal, vaginal, oral), sexual orientation, number of partners, number of new partners, prior STIs], which were adapted from other HIV/STI risk assessment tools (11, 14, 15). Table 1 displays the assigned value to each question with final scores ranging from 0 to 10 (0–3 we defined as a patient at low-risk of acquiring an HIV infection, whereas 4–10 we defined as a patient at medium-to-high risk of acquiring HIV infection).

**Table 1:** Sexual history questionnaire.

Eligibility Questions	Answers	Eligibility
Have you ever had vaginal sexual intercourse?	Yes	Eligible
	No	Ineligible
Have you had vaginal sexual intercourse in the past 3 months?	Yes	Eligible
	No	Ineligible
Are you taking or using anything to prevent pregnancy?	Yes	Eligible
	No	Eligible
Have you ever had oral sex?	Yes	Eligible
	No	Eligible
Have you ever had anal sex?	Yes	Eligible
	No	Eligible
Sexual preference—who do you have sex with? (check):	Men exclusively	Eligible
	Women exclusively	Ineligible
	Both men and women	Eligible
	Any gender, non-binary	Eligible
Scored HIV Risk Assessment Questions	Answers	Points^a^
Are you <= 25 years old?	Yes	1
	No	0
How many partners do you currently have?	0	0
	1	0
	2	1
How many sex partners have you had in the last 3 months?	0–1	0
	2–4	1
	5–9	2
	10 or more	3
Have you had a new sex partner or multiple partners within the last 3 months?	Yes	1
	No	0
When you have sex, do you use condoms?	Never	3
	Sometimes	3
	Always	0
Have you ever been told you had, or been treated for, a sexually transmitted infection?	Yes^b^	1
	No	0
Unscored HIV Risk Assessment Questions		
Have you ever had an HIV test?	Yes	
	No	
Do you have sex with a partner who injects illegal drugs?	No	
	Yes, within the past 6 months	
	Yes, more than 6 months ago	
Have you had sex with a partner who has sex with both men and women?	Never	
	Yes, within the past 6 months	
	Yes, more than 6 months ago	
Have you had sex for money, drugs, or other payment?	Never	
	Yes, within the past 6 months	
	Yes, more than 6 months ago	
Have you had sex with a partner infected with HIV?	Never	
	Yes, within the past 6 months	
	Yes, more than 6 months ago	

^a^ Low Risk Score = 0 –3; Medium/High Risk Score = 4–10 or any affirmative “Yes, within the past 6 months” response to unscored questions.

^b^ If Yes to an STI, then a selection of options are displayed. If the patient selects “HIV”, then she will be ineligible.

**Figure 2:**
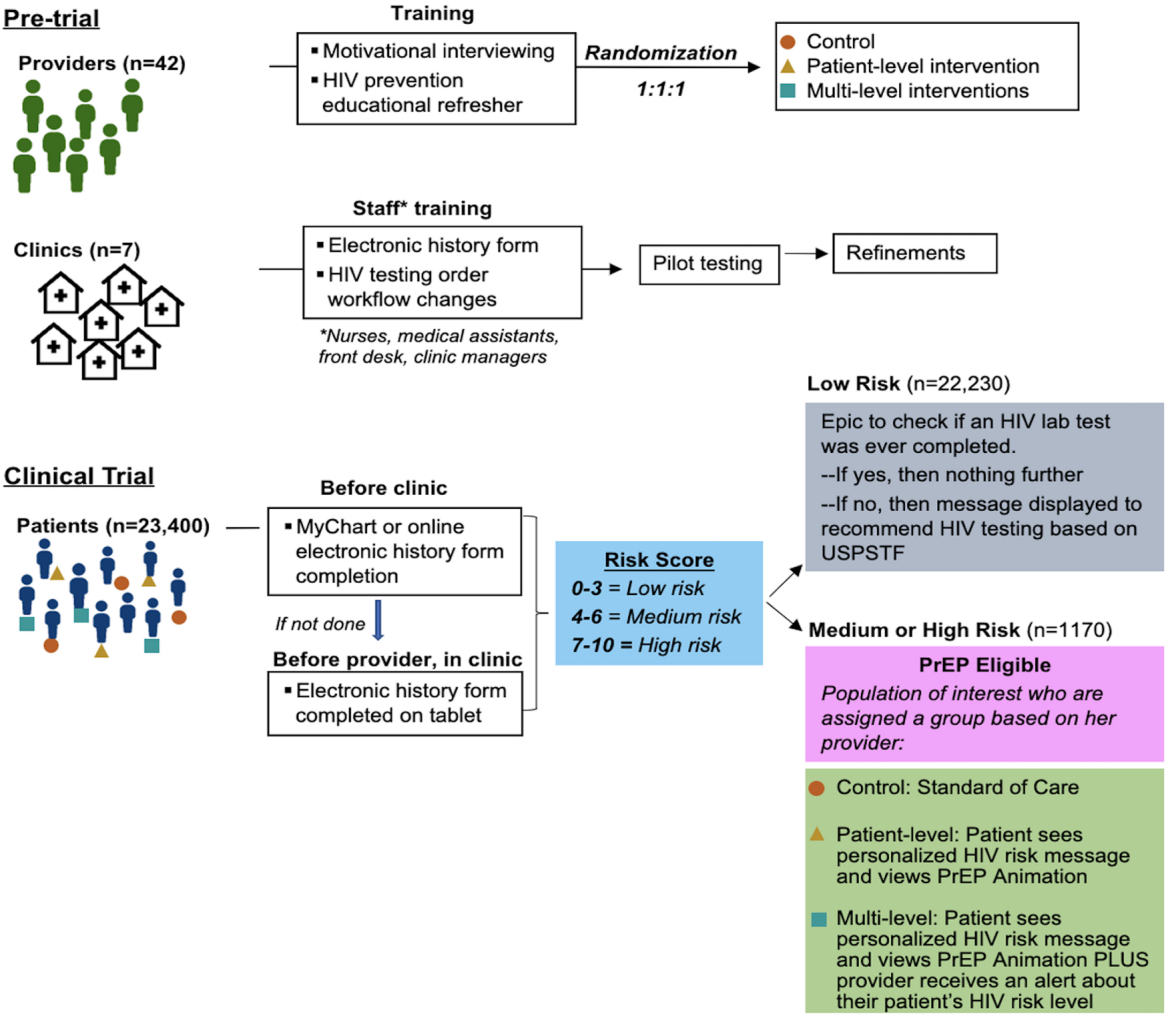
Study schema. There are two phases in the project. The first is to prepare documents, train staff, enroll providers and clinics. The second includes the clinical trial.

Patients who score 0–3 in any arm will only be offered an HIV test per USPSTF guidelines (14, 16), whereas those who score 4–10 will be considered PrEP-eligible and entered into the trial. These PrEP-eligible patients will then undergo intervention based on their provider's randomization. If their provider was randomized to arm 2, then the patient will be given a personalized message based on their sexual history questionnaire responses (Table 2) and view a 2.5-minute PrEP animation. Conversely, if their provider was randomized to arm 3, then patients will receive similar interventions to those in arm 2, but with the addition of the providers being sent a best practice alert (BPA) via the EHR advising them to offer PrEP (Figure 2).

**Table 2:** Personalized messages patients receive after questionnaire.

Risk Score Range	Tailored Message
0–3	*“The United States Preventive Services Task Force (USPSTF) recommends that individuals between the ages of 15 and 65 get tested for HIV at least once as part of routine health care and those with risk factors get tested more frequently. A general rule for those with risk factors is to get tested at least annually*.
	*Would you like an HIV test today?”*
4–10 or any affirmative “Yes, within the past 6 months” response to unscored questions	*“PrEP, or pre-exposure prophylaxis, is a medication that helps you stay HIV-negative. There are many people living with HIV in Baltimore and getting HIV is not always about your sexual behaviors. Sometimes, women are more likely to get HIV because of what's going on in their community. For that reason, you might want to consider taking PrEP in addition to other things you do for your sexual health. You can use PrEP alone or in combination with other HIV prevention methods like condoms to take care of yourself on your own terms.”*
	*“Please click here to view a short animation to learn more about PrEP.”*

### Data collection

Patient-level data and risk scores will be extracted by study staff and stored on a secured analytic framework environment, with access granted to designated study staff. To better identify barriers and facilitators to the intervention or implementation strategy, we will also conduct focus group discussions during clinic staff meetings and semi-structured, 45-minute in-depth interviews (IDIs) among interested clinic staff, providers, and patients. The interviews will include approximately 10 patients from each sexual history questionnaire score category (*n* = 30, low, medium, and high), 10 providers, and 10 members of the clinic staff. Participants will be compensated for their time and participation.

**Figure 3:**
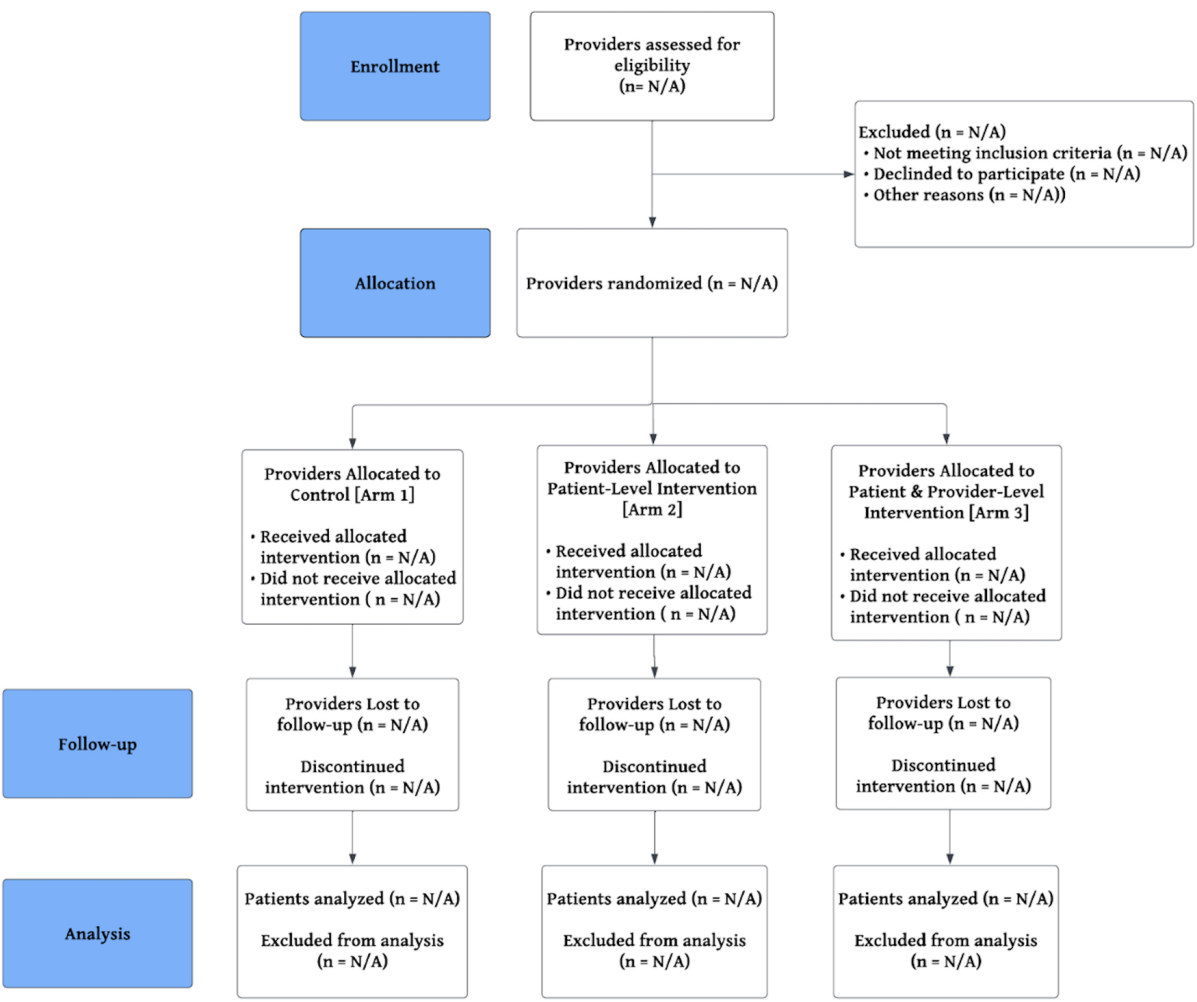
CONSORT cluster randomized trial flow diagram.

An outlined timeline for execution of provider recruitment, pre-trial training, pilot testing, data collection, and results analysis is detailed in the SPIRIT table (Table 3) below. As this study is a cluster-randomized trial, a proposed CONSORT flow diagram is included which will depict the status of provider enrollment and their exposure to allotted interventions (Figure 3). SPIRIT, CONSORT, and TIDIeR compliant checklists are provided in Supplementary Additional Files S1–S3, respectively.

**Table 3:** SPIRIT flow table.

STUDY PERIOD																							
	Enrollment	Allocation	Post-allocation																				Close-out
TIMEPOINT	*Pre-Intervention (3 M)*	Time 0	*Year 1*				*Year 2*				*Year 3*				*Year 4*				*Year 5*				*Review & Analysis (6 M)*
			*1*	*2*	*3*	*4*	*1*	*2*	*3*	*4*	*1*	*2*	*3*	*4*	*1*	*2*	*3*	*4*	*1*	*2*	*3*	*4*	
ENROLLMENT																							
Eligibility screen	x																						
Informed consent	x																						
*MI lecture*	x																						
*Digitization of EHR HIV risk tool*	x																						
Allocation		x																					
INTERVENTIONS:																							
*Eligible patients of providers receive risk assessment*			x	x	x	x	x	x	x	x	x	x	x	x	x	x	x	x	x	x	x	x	
*Provider MI learning modules*						x				x				x				x				x	
*IDIs with study participants*							X				x				x				x				x
*PrEP information sessions*	X		x	x	x																		
ASSESSMENTS:																							
*Quantitative Data: Number of PrEP prescriptions 4 weeks after visit*			x	x	x	x	x	x	x	x	x	x	x	x	x	x	x	x	x	x	x	x	x
*Number of HIV tests completed 4 weeks after visit*			x	x	x	x	x	x	x	x	x	x	x	x	x	x	x	x	x	x	x	x	x
*Number of completed questionnaires from eligible patients*			x	x	x	x	x	x	x	x	x	x	x	x	x	x	x	x	x	x	x	x	x
*ROI analysis*																							x
*Fidelity & CEA/Modeling*				x		x		x		x		x		x		x		x		x		x	x

MI, Motivational interviewing; EHR, Electronic health records; IDI, In-depth interview; PrEP, Pre-exposure prophylaxis; ROI, Return on investment; CEA, Cost-Effectiveness Analysis.

### Randomization

All eligible and consenting providers will be randomized to one of the three research arms based on restricted randomization using methods developed by Sismanidis and Moulton, et al. (17), such that clinic site, patient volume, and the prevalence of bacterial STIs (i.e., syphilis, gonorrhea, and chlamydia) in the patient population for each arm will be similar. This restricted randomization approach reduces covariate imbalances by enumerating a large set of possible allocations where balance among the above factors will be within acceptable limits, and then selecting one allocation at random using a random number generator. These limits will be specified for clinic sites such that the difference between the number of providers in any site will be no more than two, and for patient volume and prevalence of bacterial STIs such that the variability among arms will be within 10%. We will examine at least 1 × 10E19 possible allocations to determine which will meet our balance criteria. We will examine the validity matrix (the probability of being in the same arm for all provider pairs) for the balanced replications to determine if they are acceptable (18). Should some recruited providers withdraw from the study during its duration, they will be replaced with new providers. New providers who agree to participate in the study will be recruited and allocated to the intervention arms based on a block-randomized schedule with a block size of three, stratified by site. IRB-approved research coordinators and assistants blinded to the study will enroll and consent the providers and a co-investigator who is blinded to provider identification will generate the allocation sequence using R version 4.2.2 or other equivalent statistical software. Allocation concealment will be ensured as the person in charge of randomization will not be involved in the recruitment of providers, nor release the randomization sequence to any participants or research team members.

### Ethical & regulatory considerations

The Johns Hopkins Medicine Institutional Review Board (IRB) approved the study. The approval letter, and a copy of the approved study protocol, can be found in Supplementary Additional Files 4 & 5, respectively. Informed consent will be obtained from all providers and participants for all components of the study. A waiver of written informed consent was granted for the patients of the enrolled providers as the study was deemed minimal risk for three main reasons. First, the probability and magnitude of harm or discomfort anticipated in the research were not deemed greater than those ordinarily encountered in a routine clinical evaluation. The main risks to patients include breach of confidentiality and the possible risk of change from the standard of care (e.g., possible display of a personalized HIV risk message). Second, many of the HIV risk assessment questions are routine components of a comprehensive sexual history that national organizations [i.e., CDC and American College of Obstetricians and Gynecologists (ACOG)] recommend (19, 20). Third, all the methods planned for use in this study are currently accepted, recommended, and utilized in clinical visits within our health system.

### Sample size estimate

A priori sample size calculation for the three-arm randomized control trial (RCT) was based on the expected annual patient volume across seven clinics of approximately 9,100. We made the conservative assumption that 5%–10% of these will score medium or high-risk on the sexual history questionnaire based on our preliminary studies (21), yielding at least 390 patients annually, with a total of 1,170 over the three-year enrollment period. Our estimate is conservative as we found that close to 70% of adolescent and young adult women in one of our clinics scored medium or high, but these were adolescents and young adult women with the highest prevalence of STIs and risk behaviors (14). Given that patients using the same provider are not likely to be strongly related to each other in their choices for PrEP initiation (our primary outcome), we assumed a low intra-provider correlation (IPC) of 0.01, with on average 28 patients per provider per year, yielding a design effect of 1 + (28–1) * IPC = 1.27. Given these assumptions, if we estimate PrEP initiation at 6% (preliminary data) (15), we can detect a difference of 9% (from 6 to 15%) between arms with 80% power. We will examine the intervention effect within specific race/ethnicity groups. Assuming we have approximately 50% non-Latinx Black patient participants (as we do for the general patient population), we expect to enroll 585 non-Latinx Black patients, allowing for 80% power to detect a difference of 6% in PrEP initiation in the standard of care arm to 15% in the intervention arms, assuming the same design effect of 1.27.

### Outcomes & measures

We will use the Reach-Effectiveness-Adoption-Implementation-Maintenance (RE-AIM) framework to guide our analysis plan (Table 4). The primary effectiveness outcome is PrEP initiation, measured by the proportion of PrEP prescriptions written within 4 weeks of the patient's clinic visit. The primary implementation strategy outcome is sexual history questionnaire completion, measured by the proportion of patients who have a completed questionnaire in the EHR. Secondary outcomes for this study are HIV testing, comprehensive economic analyses, and fidelity measurements to determine implementation feasibility.

**Table 4:** Intervention and Implementation quantitative and qualitative outcomes using the RE-AIM framework.

	Intervention Outcomes	Implementation Strategy Outcomes	Level	Data Source
REACH	Number of patients with an eligible clinic visit who complete electronic screening assessments	Number of screening assessments that are completed electronically either via portal or in-clinic on tablet	Patient	Appointment scheduling log; EHR-programmed data transferred directly into RedCap database
	Number of patients who were excluded		Patient and Clinic	Appointment scheduling log; Observations, field notes
	Number of patients who view the animation		Patient	EHR-programmed data transferred directly into RedCap database
	*QUALITATIVE: What strategies do you think worked best to capture eligible patients? What could the clinical staff and providers have done better to reach more people?*			
EFFECTIVENESS	Number of patients who initiate PrEP (primary outcome)	Number of providers who document offering of PrEP	Patient and Providers	EHR chart review
		Number of PrEP prescriptions written	Providers	EHR discrete data element
	Number of patients who continue PrEP at 3-, 6-, 9-mos	Number of PrEP prescriptions written	Providers	EHR discrete data element
	Number of patients who are tested for HIV	Number of providers who document offering of HIV test	Patient and Providers	EHR lab data for HIV testing; EHR chart review
	*QUALITATIVE: What surprised you about the outcomes of the training, counseling, clinical care and/or treatment you received? How would you like to see the data presented?*			
ADOPTION		Number of clinics that use electronic screening assessments	Clinic	Appointment scheduling log; RedCap database; Stakeholder feedback
		Number of clinics that provide tablets to patients		
		Number of clinics that assist patients to complete electronic screening assessments		
	*QUALITATIVE: What could the team have done better to reach and recruit more providers? What are your perceptions of the training that was offered?*			
IMPLEMENTATION		Number of providers who decline the recommendations included in the alert	Provider	EHR-programmed data transferred directly into RedCap database
		Number of clinics that complete assessments in other EHR locations that are not a part of this project	Clinic	EHR chart review
				Observations, field notes
				Checklists
	*QUALITATIVE: Do you think the interventions were delivered according to plan? What costs (including time and burden, not just money) need to be considered?*			
MAINTENANCE	Above measures at 6 months after sample size achieved and research staff no longer providing support		Provider	EHR-programmed data transferred into RedCap database; chart review
			Clinic	
	*QUALITATIVE: What is the likelihood that you would continue to support this intervention? What do you think would be needed for other women, providers, or staff to find this intervention meaningful?*			

### Quantitative data analysis

We will examine whether randomization to the three intervention arms resulted in similar provider and patient groups by comparing patient and provider-level demographics with descriptive statistics. Comparison of PrEP initiation (primary outcome) between arms will be conducted based on provider arm assignment, in accordance with the intention to treat. We will use generalized linear mixed-effect models (GLMM) with logistic regression to examine the difference in PrEP initiation across arms (15). This model will allow us to consider within-and across-participant variability, while also controlling for random variables to form a multivariate normal distribution (15, 22). More specifically, we will use this model to adjust PrEP initiation comparisons for any demographic differences between arms and examine the primary outcome stratified by race/ethnicity. In exploratory analyses, we will determine whether intervention effects attenuate over time, as providers and patients may become accustomed to the intervention.

Similar to our methods for comparing the primary outcome between providers, we will also compare the rate of HIV test completion with generalized linear mixed-effect models with provider and patient-level random effects. We will consider adjusting for imbalanced factors between arms in these analyses. In addition, we will also examine PrEP initiation among low risk scoring patients to determine whether providers receiving the intervention are more generally influenced in their behavior or if the intervention effect is specific only to intervention patients. All questionnaire data will be collected via the EHR, and the R programing language (version 4.1) will be used for analyses. Datasets generated and analyzed during this study will be made publicly available upon publication of results in an institutional repository.

### Qualitative data analysis

We will conduct an explanatory sequential design because the quantitative study is conducted first, followed by a qualitative evaluation to explain or expound on the quantitative findings (23). Certain quantitative measures (e.g., demographics, satisfaction rates) will be imported into the Dedoose® database. Simple inferential statistics for two-way tables (e.g., Chi-square) will be used to evaluate thematic differences between groups by site, demographics, and other critical predictors identified in the quantitative analyses. Demographic and clinical data will be used to determine associations between survey responses and demographic variables.

### Economic evaluation

We will first conduct a cost-effectiveness analysis (CEA) from the perspectives of a payer or an adopting organization (e.g., Medicaid), to evaluate the cost and benefit tradeoffs of adopting alternative intervention strategies. We will use standard analytical methods recommended by the Second Panel on Cost-Effectiveness Analysis (Table 5) to estimate the effectiveness and economic costs of each active intervention (i.e., patient-focused intervention and patient-focused intervention plus EHR alerts) and standard of care (24–27). Costs will be estimated from the perspective of an adopting organization or payer (e.g., Medicaid), and will include both program implementation costs, the direct medical costs related to STI testing, treatment and counseling, and costs associated with other medical care resource use, over the observation period (1 year). We will exclude all research-related costs, such as those related to the implementation of the trial itself (e.g., research staff time). A micro-costing approach will be used to track all major and relevant resource items used to implement the intervention and their associated unit costs over a one-year period (28, 29). Individual resource item costs will be calculated by multiplying the units of resource use with the unit costs of each resource item. We will combine these costs to calculate the total cost of implementing each intervention. The implementation costs of home-based STI testing will be defined to include those associated with resources used for specimen collection for STI testing and retesting, STI counseling and treatment and partner therapy, as well as for the monetary and non-monetary (i.e., electronic vouchers) incentives, logistics (e.g., transportation), and the administration and delivery of each intervention. We will track the units of STI tests and STI counseling sessions, medication prescriptions, clinical and non-clinical staff time, and medical care visits. The unit costs of STI tests and counseling sessions will be derived from the existing literature, cost charts, and fee schedules. Clinical staff time will be defined to include doctor, nurse, and STI counselor time spent with the patient; non-clinical staff time will be defined as any other staff time spent on the patient. We will record staff time in minutes used by the study's staff (both clinical and non-clinical) to conduct the program's activity. We will use the average salaries for staff to estimate an average unit cost per encounter. Staff salaries will be obtained from the published list of public sector salaries for specific occupations. We will combine these costs to calculate the total cost of implementing each intervention.

**Table 5:** Impact inventory, adapted from sanders et al., 2016 (24).

Sector	Type of Impact (list category within each sector with unit of measure if relevant)^a^	Included in This Reference Case Analysis From…Perspective?		Notes on Sources of Evidence
		Health Care Sector	Societal	
Formal Health Care Sector				
Health	Health outcomes (effects)			
	Longevity effects	☑	☑	
	Health-related quality-of-life effects	☑	☑	
	Other health effects (eg, adverse events and secondary transmissions of infections)	☑	☑	
	Medical costs			
	Paid for by third-party payers	☑	☑	
	Paid for by patients out-of-pocket	☑	☑	
	Future related medical costs (payers and patients)	☑	☑	
	Future unrelated medical costs (payers and patients)	☑	☑	
Informal Health Care Sector				
Health	Patient-time costs	NA	☑	
	Unpaid caregiver-time costs	NA	□	
	Transportation costs	NA	☑	
Non-Health Care Sectors (with examples of possible items)				
Productivity	Labor market earnings lost	NA	☑	
	Cost of unpaid lost productivity due to illness	NA	☑	
	Cost of uncompensated household production^b^	NA	□	
Consumption	Future consumption unrelated to health	NA	□	
Social Services	Cost of social services as part of intervention	NA	☑	
Legal or Criminal Justice	Number of crimes related to intervention	NA	□	
	Cost of crimes related to intervention	NA	□	
Education	Impact of intervention on educational achievement of population	NA	□	
Housing	Cost of intervention on home improvements (eg, removing lead paint)	NA	□	
Environment	Production of toxic waste pollution by intervention	NA	□	
Other (specify)	Other impacts	NA	□	

NA indicates not applicable.

^a^ Categories listed are intended as examples for analysts.

^b^ Examples include activities such as food preparation, cooking, and clean up in the household; household management; shopping; obtaining services; and travel related to household activity (25).

Direct medical costs will include those associated with laboratory tests, health facility visits for STIs, and medications for STI treatment. These costs will be calculated by multiplying the amounts of resources used by their unit costs. We will track direct medical resource uses through our micro-costing approach. The unit costs associated with physician visits for specific diagnoses, laboratory tests and drugs will be derived from various fee schedules (30). Direct non-medical costs (e.g., patient transportation costs), indirect costs (e.g., lost productivity), and intangible costs (e.g., psychosocial costs or pain and suffering) will be excluded from this study, given the perspective adopted. We will combine the implementation costs and direct medical costs to calculate the total costs associated with each experimental arm.

All costs and benefits will be discounted at the conventional 3% annual discount rate. As the value of health effects of prevention tends to increase over time (e.g., each HIV infection detected or each new woman treated with PrEP may avert new HIV infections) (31), we will also conduct robustness analyses with higher discount rates for the health benefits. In addition, we will adjust for inflation by indexing all prices to a single year's prices (e.g., year 2023 prices), using consumer price indices from the US Bureau of Labor Statistics.

Using the estimated effectiveness and costs above, we will calculate the relative efficiency of each active intervention (i.e., patient-focused intervention and patient-focused intervention plus EHR alerts) relative to standard of care in terms of their incremental cost-effectiveness ratios (ICER), calculated as the ratio of each intervention's incremental costs to its incremental benefits (e.g., incremental new STI/HIV test, new STI/HIV case diagnosed, number of new women on PrEP), and expressed in terms of dollars per health benefits. The ICER will be compared to a payer's “opportunity cost,” i.e., willingness-to-pay (WTP) threshold, to determine the intervention's acceptability in terms of its efficiency in generating health benefits and under the implementation strategy. An ICER below the WTP threshold will be indicative of good value for money produced by the intervention. We will also calculate the net and incremental monetary and health benefits of alternative interventions.

To characterize uncertainty's impact on the recommended alternatives' robustness, we will first conduct deterministic and probabilistic sensitivity analyses (DSA and PSA). These analyses will help identify the most influential measures (DSA) and assess the joint effects of uncertainties in all cost and effectiveness measures on the distributions of the ICER (PSA). Values generated by the multivariate PSA will enable us to construct credible simulation intervals around key outcome measures, and to estimate the cost-effectiveness acceptability curves (CEAC) and frontier (CEAF) (32–36), the expected loss curves (ELCs), and the cost-effectiveness risk-aversion curves (CERAC) (37).

Second, a budget impact analysis will be conducted to estimate the affordability of adopting alternative interventions. Cost-effectiveness affordability curves (CEAFC) will be constructed to characterize both dimensions of the joint distribution of incremental costs and effects on the cost-effectiveness plane (38).

Third, we will conduct a return-on-investment (ROI) analysis to quantify the profitability of alternative interventions.

Last, we will apply the innovative method of distributional cost-effectiveness analysis (DCEA) to quantify the health equity impact of alternative interventions and implementation strategies, and to assess potential tradeoffs between their costs, health benefits, and equity impacts across women in different race/ethnic groups (e.g., White, Black, Hispanic women) (39).

We will measure health equity impact in terms of the equally distributed equivalent (EDE) value of the incremental net health benefit (INHB), which adjusts the INHB to account for equity considerations, using a social welfare evaluation function (SWF) such as the Gini, the Atkinson-Sen, and Kolm-Polack SWF (40, 41). The EDE value represents the level of the effectiveness outcome that, if provided uniformly to every group in a population, would yield the same amount of social welfare to the distribution of that outcome (40).

Results will be presented in the equity impact plane, which captures potential trade-offs between the net equity impact and the net health impacts of alternative interventions. Efficiency-equity tradeoff curves will also be constructed to convey the worthiness of each intervention to the payer or decision-maker at different levels of aversion to inequality and WTP threshold.

## Discussion

Our study will focus on three of the four pillars of the EHE in the U.S.—diagnosing HIV with immediate linkage to treatment services, and preventing new HIV infection with PrEP initiation—targeting OB/GYN providers and their patients (42). Our hybrid type II effectiveness-implementation trial seeks to prevent new HIV transmissions by increasing patient awareness of HIV risk factors and educating at-risk patients about prevention strategies, inclusive of PrEP. Furthermore, increasing HIV testing among our patients should make a lasting impact on the clinical care of U.S. cisgender women.

The strengths of our study include the exclusive focus on U.S. cisgender women, who currently are not the target of most HIV prevention studies in the U.S. Second, we created interventions and an implementation strategy that addressed commonly cited patient- and provider-level barriers to PrEP initiation and HIV testing. Some of the interventions and strategies include utilizing EHR automation to decrease provider burden and digitizing a sexual history questionnaire to remove discomfort with asking or answering questions related to sexual history and behaviors. Third, we targeted OB/GYN providers who routinely discuss sexual behaviors and STIs and appear to be ideal prescribers of PrEP for women. Fourth, we apply a battery of economic evaluation analyses to quantify the value for money of the alternative interventions, to better inform decision-making and guide sustainability efforts. However, there are limitations. First, our HIV efforts will only impact women who are engaged in clinical care. However, we included two Federally Qualified Health Centers, which are safety net clinics that provide care to patients regardless of insurance coverage. Next,our study will only be generalizable to clinics with EHRs, which are costly systems. Lastly, the primary outcome measure of the number of PrEP prescriptions ordered will not necessarily equate to the medications received and taken by the targeted patient. However, when possible, we will determine whether the prescription had been filled and medication taken.

In sum, our novel multimodal, multilevel implementation science protocol seeks to test the feasibility, acceptability, and efficacy of a scored digital sexual health questionnaire intervention and EHR implementation strategy to increase HIV prevention strategies and PrEP initiation among U.S. cisgender women during routine OB/GYN clinics.

## Ethics statement

The studies involving human participants were reviewed and approved by The Johns Hopkins Medicine Office of Human Subjects Research Institutional Review Boards. Written informed consent from the participants' legal guardian/next of kin was not required to participate in this study in accordance with the national legislation and the institutional requirements.

## Supplementary material

**SUPPLEMENTARY ADDITIONAL FILE S1**

entails the SPIRIT compliant checklist for this randomized trial.

**SUPPLEMENTARY ADDITIONAL FILE S2**

entails the CONSORT compliant checklist extension for this cluster-randomized trial.

**SUPPLEMENTARY ADDITIONAL FILE S3**

entails the TIDIeR compliant checklist for the interventions in this study.

**SUPPLEMENTARY ADDITIONAL FILE S4**

entails the approval letter received by our ethics committee to conduct this trial.

**SUPPLEMENTARY ADDITIONAL FILE S5**

entails the original study protocol reviewed and approved by our ethics committee.
