# Supplementary material for: An effectiveness-implementation trial protocol to evaluate PrEP initiation among U.S. cisgender women using eHealth tools vs. standard care
Source: Front Reprod Health. 2023 Jun 8;5:1196392. doi: 10.3389/frph.2023.1196392 (PMC10285440; doi:10.3389/frph.2023.1196392)
Supplement: Supplementary file 1 [file Datasheet1.pdf]

Clinic-based HIV Identification and Prevention Project using Electronic Resources (CHIPPER)
Version 1.x (Dated July 25, 2022)

Sponsored by:

National Institute of Mental Health
US National Institutes of Health

IND#: None

Principal Investigator:
Jenell Coleman, MD, MPH
Johns Hopkins University School of Medicine
Baltimore, MD USA

Version 1.x
July 25, 2022

TABLE OF CONTENTS	Page
List of Abbreviations and Acronyms	4
Protocol Team Roster	5
Protocol Summary	7
**1. KEY ROLES**	**8**
**2. INTRODUCTION**	**9**
Background Information	9
Study Hypothesis and Rationale	10
**3. OBJECTIVES**	**10**
Primary Objectives	10
Secondary Objectives	10
**4. STUDY DESIGN**	**10**
**5. STUDY POPULATION**	**13**
Selection of the Study Population	13
Participant Inclusion Criteria	13
Participant Exclusion Criteria	13
**6. STUDY PRODUCT/INTERVENTION(S)**	**14**
Assessment of Adherence to Study Intervention(s)	14
**7. STUDY PROCEDURES/EVALUATIONS**	**15**
Clinical Evaluations and Procedures	15
Fidelity	16
**8. STATISTICAL CONSIDERATIONS**	**16**
Study Endpoints	17
Sample Size Considerations	17
Randomization and Blinding	17
Data Analysis Plan	17
**9. ASSESSMENT OF SAFETY**	**17**
Definition of an Adverse Event (AE)	17
Adverse Event Procedures and Reporting Requirements	19
Serious Adverse Events	19
Grading Severity of Events	20
**10. CLINICAL MANAGEMENT**	**20**
HIV Test Management	20
Social Harms	20
Criteria for Temporary or Permanent Discontinuation of Study Product	20
Criteria for Early Termination of Study Participation	20
Pregnancy	20
**11. DATA AND SAFETY MONITORING**	**21**
**12. DATA HANDLING AND RECORDKEEPING**	**21**
Investigator's Records	21
Data Integrity	21
**13. HUMAN SUBJECTS PROTECTIONS**	**22**
Potential Risks	22
Protection Against Risks	22
Benefits	22
Informed Consent Process	22
Waiver of Informed Consent	23
Importance of Knowledge to be Gained	25
Payment and Remuneration	25
Costs	26
**14. Laboratory Evaluations**	**26**
Laboratory Evaluations and Specimen Collection	26

Specimen Storage and Future Use	**26**
Biohazard Containment	**26**
**15. REFERENCES**	**27**
**16. APPENDICES**	
APPENDIX A: Paper Patient History Form	
APPENDIX B: Existing Epic Assessments and Alerts	
APPENDIX C: Gyn Risk Tool	
APPENDIX D: Coordinating Center Summary	

i

LIST OF ABBREVIATIONS AND ACRONYMS

AE	Adverse experience/event
AIDS	Acquired Immunodeficiency Syndrome
CDC	Centers for Disease Control and Prevention
CLIA	Clinical Laboratory Improvement Amendments
CRF	Case report form
CRU	Clinical Research Unit
DSMB	Data safety and monitoring board
EAE	Expedited Adverse Event
EHR	Electronic Health Record
ESHxForm	Electronic History Form
FDA	(United States) Food and Drug Administration
FTC	Emtricitabine
GCP	Good Clinical Practices
Gyn	Gynecology
HIV	Human Immunodeficiency Virus
ICH	International Committee on Harmonisation
IP/CP	Integrated Preclinical/Clinical Program
IRB	Institutional Review Board
JHHWHP	Johns Hopkins Hospital Women's HIV Program
JHU	Johns Hopkins University
MSM	Men who have Sex with Men
NIMH	(United States) National Institute of Mental Health
NIH	(United States) National Institutes of Health
ObGyn	Obstetrics and gynecology
PrEP	Pre-exposure prophylaxis
PID	Participant Identification Number
PRN	As needed
QD	Daily
SAE	Serious Adverse Experience
SADR	Suspected Adverse Drug Reaction
SOE	Schedule of Events
SSP	Study-specific procedures
STD	Sexually Transmitted Disease
STI	Sexually Transmitted Infection
SUSAR	Suspected, Unexpected Serious Adverse Reactions
TDF	Tenofovir disoproxil fumarate
TFV	Tenofovir

PROTOCOL TEAM ROSTER

Johns Hopkins Team:
Jenell Coleman, MD, MPH
Principal Investigator
Associate Professor of Gynecology/Obstetrics
Department of Gynecology and Obstetrics
Johns Hopkins University School of Medicine
600 North Wolfe Street, Phipps 249
Baltimore, MD 21287
Telephone: 410-614-4496
Fax: 410-955-1003
Email: colemanj@jhmi.edu

Stephen Martin, MD
Co-investigator
Section Chief, JHCP Gynecology and Obstetrics
Instructor Department of Gynecology and Obstetrics
Baltimore, MD 21224
Telephone: 410-522-9940
Email: smarti99@jhmi.edu

Emmanuel Drabo, PhD
Economist
Assistant Professor of Health Policy & Management
Bloomberg School of Public Health
Baltimore, MD 21224
Telephone: 410-502-5127
Email: edrabo@jhu.edu

Jamie Perin, PhD
Statistician
Biostatistics, Epidemiology, and Data Management (BEAD) Core Faculty Lead
Associate Scientist, Global Disease Epidemiology and Control
Baltimore, MD 21287
Telephone: 410-955-3906
Email: jperin@jhu.edu

Study Coordinator
TBD

Implementation Partner Team (JHHWHP):
Jean Anderson, MD
Consultant
Baltimore MD 21287
Telephone: 410-614-4496
Email: janders@jhmi.edu

Rosemary Ramroop
HIV Counselor and Tester, Community Outreach Specialist
Baltimore MD 21287
Telephone: 410-614-3023
Email: rramroo1@jhmi.edu

<u>University of Michigan Site (FWA 00004969)</u>
Okeoma Mmeje, MD MPH
Principal Investigator
Assistant Professor of Obstetrics and Gynecology
University of Michigan Medical School
Telephone: 734-763-3429
Email: ommeje@med.umich.edu

Golfo Tzilos Wernette, PhD
Co-Investigator
Associate Professor of Family Medicine and Psychiatry
University of Michigan Medical School
Telephone: **734-998-7874**
Email: gtzilos@med.umich.edu

<u>NIH</u>
Christopher Gordon, PhD
NIMH Program Officer
Rockville, MD, 20852
Phone: 240-627-3867
Email: cgordon1@mail.nih.gov

PROTOCOL SUMMARY

**TITLE:**	**Clinic-based HIV Identification and Prevention Project using Electronic Resources (CHIPPER)**
**PRINCIPAL INVESTIGATORS:**	Jenell Coleman, MD MPH (Administrative), Okeoma Mmeje, MD MPH
**SAMPLE SIZE:**	N=23,492 (Ob/Gyn clinic providers N=42, Patients N=23,400, Staff =50)
**STUDY POPULATION:**	Ob/Gyn providers, patients, and Hopkins University clinic staff
**ENROLLING SITE:**	Johns Hopkins University Sch of Medicine ObGyn Clinics, Baltimore
**STUDY DESIGN:**	Hybrid Type II Effectiveness-Implementation Trial
**STUDY DURATION:**	5 years
**STUDY SCHEMA:**	

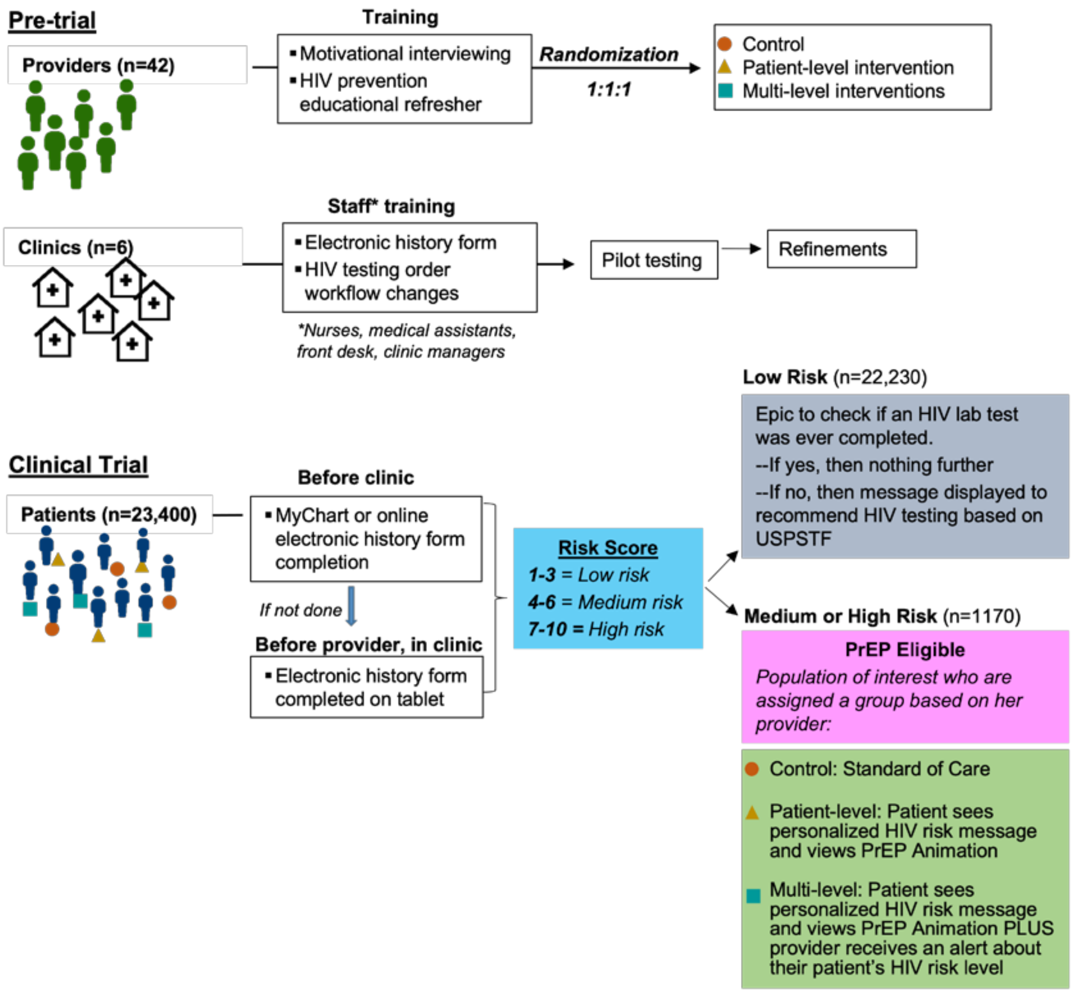

**PRIMARY OBJECTIVES:**	Effectiveness: increase PrEP uptake<br>Implementation: measure adoption of the protocol
**PRIMARY ENDPOINTS:**	Effectiveness: PrEP prescription<br>Implementation: proportion of patients who complete the electronic Gyn history form, number of providers declining automated orders

KEY ROLES

1.1 Protocol Identification

Protocol Title: **Clinic-based HIV Identification and Prevention Project using Electronic Resources**

Short Title: **CHIPPER**

Date: June 29, 2022

1.2 Funding Agency

Funding Agency: National Institute of Mental Health (NIMH)
National Institute of Health (NIH)
6700 B Rockledge Drive
Bethesda, MD 20892 USA

1.3 NIMH Program Officer

Christopher Gordon, PhD

1.4 Study Site Investigators

Principal Investigator:	Jenell Coleman, MD, MPH
Principal Investigator:	Okeoma Mmeje, MD MPH (Michigan)
Co-Investigator:	Emmanuel Drabo, PhD
Co-Investigator:	Jamie Perin, PhD
Co-Investigator:	Stephen Martin, MD
Co-Investigator:	Golfo Tzilos Wernette, PhD (Michigan)

1.5 Institutional Review Board

Johns Hopkins Medicine IRB will serve as the single IRB of record

1.6 Study Sites Scope of Work

Johns Hopkins' investigators will assume the overall administrative authority and responsibility for regulatory compliance, including reports to the NIH, IRB, and other Johns Hopkins University institutional authorities. Additionally, Hopkins' investigators will: 1) create and implement the electronic medical record changes; 2) enroll all participants; 3) conduct written and oral informed consent procedures; 4) onboard obgyn clinic sites; 5) coordinate focus group and in-depth interviews to obtain feedback from study participants, including providers, patients, and clinic staff; 6) collect field notes; 7) arrange transcription of qualitative methods; 8) quantitative data analysis, including economic analyses; 9) assure quantitative data security and integrity; and 10) write manuscript.

University of Michigan's investigators will: 1) develop the qualitative instruments (i.e., in-depth interview and focus group guides); 2) conduct the focus groups and in-depth interviews virtually; 3) analyze the qualitative data; 4) use the qualitative data to provide feedback to Hopkins' team about the intervention; 5) assure qualitative data security and integrity; 6) train Hopkins' providers to use motivational interviewing techniques; and 7) write manuscript.

2. INTRODUCTION

2.1 Background Information

Cisgender (i.e., person's gender identity corresponds with the identified sex at birth)[1] women comprise approximately one out of every five new HIV diagnoses in the United States in 2018, with 85% of cases attributed to heterosexual contact.[2] Black women are disproportionately affected, comprising 60% of new HIV infections among women, although they comprise only 14% of the female population.[2] Black women have one of the highest incidences of HIV, second only to men who have sex with men (MSM). Consistent condom use, daily oral tenofovir disoproxil fumarate 300mg-emtricitabine 200mg (TDF-FTC) as PrEP, and male partner HIV testing are evidence-based interventions for women to reduce their risk of HIV acquisition, yet many barriers to uptake and access to these strategies remain for cisgender women. Our proposal will focus on three of the four pillars of the **Ending the HIV Epidemic (EHE)** in the U.S. – diagnosing and treating HIV rapidly and preventing new HIV infection with PrEP initiation.[3]

2

PrEP use among U.S. cisgender women. TDF-FTC, the only oral Food and Drug Administration (FDA)-approved PrEP medication for women, has been authorized for use since 2012.[4] However, PrEP uptake and effectiveness requires that women be aware that such treatments exist, know how to access it, believe they are at risk for HIV infection, and adhere to the dosing regimen. PrEP awareness among women has been low since approval, partly because many PrEP marketing strategies and demonstration projects targeted MSM and transgender women.[5,6] However, recent data from an obstetrics and gynecology (ObGyn) clinic (where women receive prenatal care and sexual and reproductive health services) show that up to 44% of women have heard of PrEP.[7] Nevertheless, PrEP use remains low with only 1-6% of PrEP users being women.[8,9] Further, Black women - who have the greatest need for HIV prevention - are four times less likely to be using PrEP than White women.[10,11] Reasons for poor PrEP uptake are multi-factorial, with underestimation of HIV risk as the most commonly cited patient-level reason. In one study, 85% of ObGyn clinic patients considered themselves low-risk for HIV acquisition, despite living in an EHE priority city, thus only 41% used condoms.[7] Similarly, a survey of pregnant women demonstrated that there was no association between the number of behavioral risk factors and perceived risk of HIV acquisition.[12] In contrast, when risk perception is high (e.g., in suspected concurrent partnerships or mistrust), young Black women are more likely to use condoms and other sexually transmitted infection (STI) prevention strategies.[13] Therefore, assisting cisgender women with understanding their own HIV risk is critical to Ending the HIV Epidemic.

Provider-level reasons for poor PrEP uptake among cisgender women include their inability to accurately assess their patients' risk for HIV acquisition and lack of knowledge.[14-16] Providers (i.e., physicians, nurse practitioners, physician-assistants, and midwives) have an increasing number of concerns and issues to address during a clinical visit that is ever-decreasing in length. Providers must decide whom to screen for STIs, including HIV, which leaves the potential for under-or over-estimation of risk based on provider's judgment and bias. If providers do not assess sexual behaviors, perhaps due to discomfort or time, they may miss opportunities to provide appropriate testing or counseling.[17] The current clinical landscape and time constraints hinder identification of PrEP-eligible women, especially since an accurate risk assessment tool does not exist. Thus, providers could benefit from having an objective assessment tool to assist with identification of women who may be at-risk of HIV infection.

HIV testing of cisgender women and knowledge of their male partners' HIV status. There are missed opportunities for HIV testing of women during routine clinical visits; data show that 37-45% of persons newly diagnosed with HIV had a least one clinical visit in the prior 12 months prior to their diagnosis.[3] Furthermore, a Medicaid claims database study reported that less than 45% of patients who tested positive for gonorrhea or syphilis, biologic markers for HIV risk, were tested for HIV.[18] Our data in a pregnant population in a city with high HIV prevalence showed that although over 90% of women had at least one HIV test during pregnancy, HIV retesting was rarely performed (<30%), even among women with recurrent STIs or new sexual partner and inconsistent condom use.[19]

Further exacerbating the discordance between risk perception and actual risk is that women tend to be unaware of their male partners' HIV serostatus and HIV risk factors. Twice as many Black HIV-infected men reported having sex with both men and women compared to White HIV-infected men (34% vs 13%).[20]

Evidence-gap. An accurate and precise HIV risk assessment tool currently does not exist for U.S. cisgender women and may not ever exist because the HIV incidence in U.S. women is too low for validation studies. We posit that using biologic markers of HIV susceptibility like inflammatory STIs (e.g., gonorrhea, trichomoniasis, genital herpes, or chlamydia) could be used as a nudge to prompt providers to discuss HIV prevention (i.e., HIV testing and PrEP) with patients. STIs increase HIV susceptibility in women by increasing the population of CD4+T cells, which are the cells that are infected by HIV, recruited into the female genital tract.[21] Therefore, using a survey that could predict an STI would be useful during a clinic visit. Our group has published two manuscripts using an STI Risk Quiz,[22,23] which has been validated in several studies and could be a useful, albeit imperfect, proxy for increased HIV risk. Although the CDC updated the PrEP guidelines in 2021 and included a recommendation to inform all sexually active adults and adolescents about PrEP,[24] this information is not widely known. Furthermore, the guidelines state that people at *substantial risk of HIV* acquisition should be offered PrEP. Our project was designed to equip patients and providers with PrEP knowledge via short animations, electronic health record (EHR) decision support tools, and HIV risk level assessments.

2.2 Study Hypothesis and Rationale

Our hypothesis is that by gathering information on sensitive sexual behaviors electronically, communicating HIV risk in an understandable and relatable format to patients and ObGyn providers, and automating parts of clinical care, PrEP initiation and HIV testing will increase. ObGyn clinics in Baltimore, Maryland, which is an EHE priority area, will be targeted. Since TDF-FTC was approved, women have consistently expressed a **preference for receiving HIV prevention services in clinics where they receive ObGyn care,**[20] as opposed to specialty clinics that treat infectious diseases or STIs where most PrEP projects are located.[7,25,26] Additionally, we based our intervention and implementation strategy on the Information, Motivation, and Behavior Model (IMB) of Behavior Change (**Figure 1**).

Figure 1. Information Motivation Behavioral Model

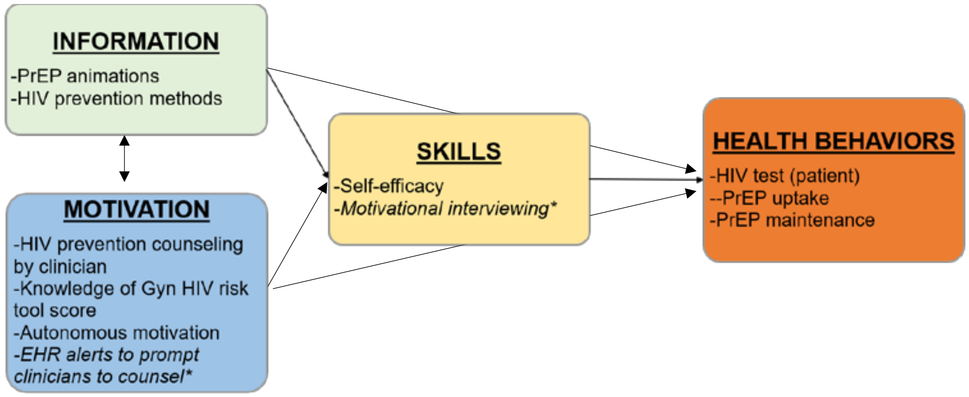

3. OBJECTIVES

3.1 Primary Objectives:

3.1.1 Intervention: to determine which intervention (patient-level only vs. patient- and provider-level) is the most effective in increasing PrEP uptake.

3.1.2 Implementation Strategy: to measure the adoption of using electronic resources (e.g., computerized Gyn HIV Risk Tool integrated into an EHR and automated HIV test orders) in each clinic.

3.2 Secondary Objectives:

3.2.1 Intervention: to increase HIV testing; to identify contextual factors, facilitations and barriers associated with PrEP uptake;

3.2.2 Implementation Strategy: comprehensive economic analyses and fidelity measurements.

4. STUDY DESIGN

This is a Hybrid Type II effectiveness-implementation trial. A type II trial was selected because we are simultaneously testing the effectiveness of our intervention and implementation strategy. In other words, we are testing an implementation strategy during an effectiveness trial.

There are a few pre-trial procedures that will take place in **Phase I** including the following:

(1) **Motivational interviewing:** ObGyn provider training will be led by co-investigator Dr. Tzilos Wernette. Dr. Golfo Tzilos Wernette, has been invited as a speaker at the Gynecology and Obstetrics Department Grand Rounds to provide one 45-50-minute training for the department. Her scheduled date is September 29, 2022.

(2) **Creation of the electronic sexual history form (eSHxForm) that contains the Gyn HIV Risk Tool**: Currently, patients who have enrolled in MyChart, which is the patient portal for Epic, are sent a "Patient Reported History" questionnaire. These questionnaires are systems-wide and tailored to the department. Additionally, some divisions have developed secondary questionnaires that are specific to their clinical care needs, which are sent to patients before the clinic visit. As part of this research project, we will create a secondary questionnaire using the sexual health portion of our current paper history form (JH GYN NPQ A (05/16) **Appendix A**). We will add four additional sexual history questions from our Epic Risk Assessment Flowsheet (**Appendix B**).

(3) **EHR changes:** (a) The new eSHxForm will be integrated into the EHR after approval from the MyChart Committee. We have met with the chair to discuss our protocol and the changes are feasible; (b) We will add a point value to each sexual history question and program Epic to summarize the score behind the scenes. Based on the score, different messages will be shown to the patient (**Appendix C**). The score will not be visible in the EHR but part of the metadata on the backend. Scoring is done for several questionnaires already and will not be problematic; (c) We will edit our current provider EHR alert (**Appendix B**) to report their patients' HIV risk assessment level (based on how patients answer the sexual health questions above) and ask if the provider would like to discuss PrEP and add the required laboratory tests before starting PrEP.

(4) **Staff training**: During scheduled staff meetings, group huddles, or individual meetings, we will discuss the eSHxForm workflow with nurses, clinic managers, front desk staff, medical assistants, and other relevant clinical staff. This will allow us to troubleshoot any anticipated problems. Patients are sent a clinic reminder text message before clinic visits. Staff will encourage patients to complete the MyChart questionnaires (general history and the secondary eSHxForm) called the "eCheck-in". This best practice is being used at some clinical sites within Obgyn already but not all.

(5) **Pilot testing**: We will pilot test the eSHxForm workflow at each clinical site to determine the most effective strategies to get patient participation before the clinic visit.

After the pre-trial procedures, enrolled providers (N=42) will be randomly assigned 1:1:1 to a study arm mentioned below.

Phase II includes conducting the clinical trial. Patients of the enrolled provider who attend the ObGyn clinic for any preventative visit will complete an electronic sexual history form (eSHxForm) before engaging the provider. Ideally, patients will complete the eSHxForm before clinic through a message sent through the patient portal (MyChart), email, or secure text messaging. If this has not been completed, then a computer tablet will be used while the patient waits for their provider.

Patients who score low (0-3) will receive an electronic message offering a laboratory HIV test per USPSTF guidelines, only if Epic does not locate an HIV test in the record already. USPSTF recommends those 15-65 receive at least one HIV test in their lifetime.[27] If she accepts, then an EHR alert will pop-up for the clinic staff to pend the HIV test order for the provider to discuss with the patient and discuss. The clinic staff will notify each provider when there is an HIV test order pending in the chart. Based on our preliminary data, we anticipate that the majority of our patients will fall into this category of low risk (>90-95%).

Patients who score medium or high (4-10) will be assigned a study arm based on their provider's group. This will occur automatically via programming integrated into the EHR based on the provider and the type of scheduled visit. The score will be recorded in EHR but it will not be labeled, i.e. a number will be there but nothing about HIV or risk of HIV. This medium/high score group is whom we consider **PrEP-eligible** *(e.g., at substantial HIV risk)* and is the focus of this implementation science study. Based on our preliminary data, we anticipate that a minority of patients (5-10%) will fall into these categories of medium or high risk.

Clinical trial arms. The Interventions are guided by the Information-Motivation-Behavior model of behavior change (**Figure 1**).
-Arm 1 Control: standard of care
-Arm 2 Patient-level intervention: patients will be shown a 2.5-minute PrEP animation (information) and their personalized HIV risk messaging (motivation)
-Arm 3 Patient and Provider multi-level intervention: patients will be shown a 2.5-minute PrEP animation (information) and their personalized HIV risk messaging (motivation) AND providers will receive an EHR alert about their patient's personalized HIV risk messaging and a recommendation to offer PrEP. The alert will allow the provider to accept to add the required laboratory tests to start PrEP or decline altogether. Orders will have to be signed by providers but the correct laboratory tests will be ordered to save time and ensure accuracy.

There is no difference between Arms 2 and 3 regarding information and motivation. The difference between these two arms is the provider activity. We are trying to tease apart a clinic-based intervention that targets patients alone (Arm 2) versus both patients and providers (Arm 3). The primary outcome (behavior) is PrEP uptake, and this will be compared based on the Arm the patient is assigned, as is discussed in the data. analysis plan. As competing interests (e.g., lack of time) and difficulty assessing the patient's HIV risk consistently have been listed as provider-level barriers to PrEP uptake; and patients do not perceive themselves to be at-risk of HIV,[28-30] having these two arms will give us a lot of information about how to increase PrEP in women. For instance, Arm 2 will determine whether patients' awareness of their HIV risk will prompt them to ask their provider about PrEP or increase their desire to start PrEP; while Arm 3 will add an additional PrEP facilitator and determine if providers' awareness of their patients' HIV risk level will prompt them to discuss PrEP or increase PrEP uptake by patients.

Figure 2. Study Schema. There are two phases in the project. The first is to prepare documents, train staff, enroll providers and clinics. The second includes the clinical trial.

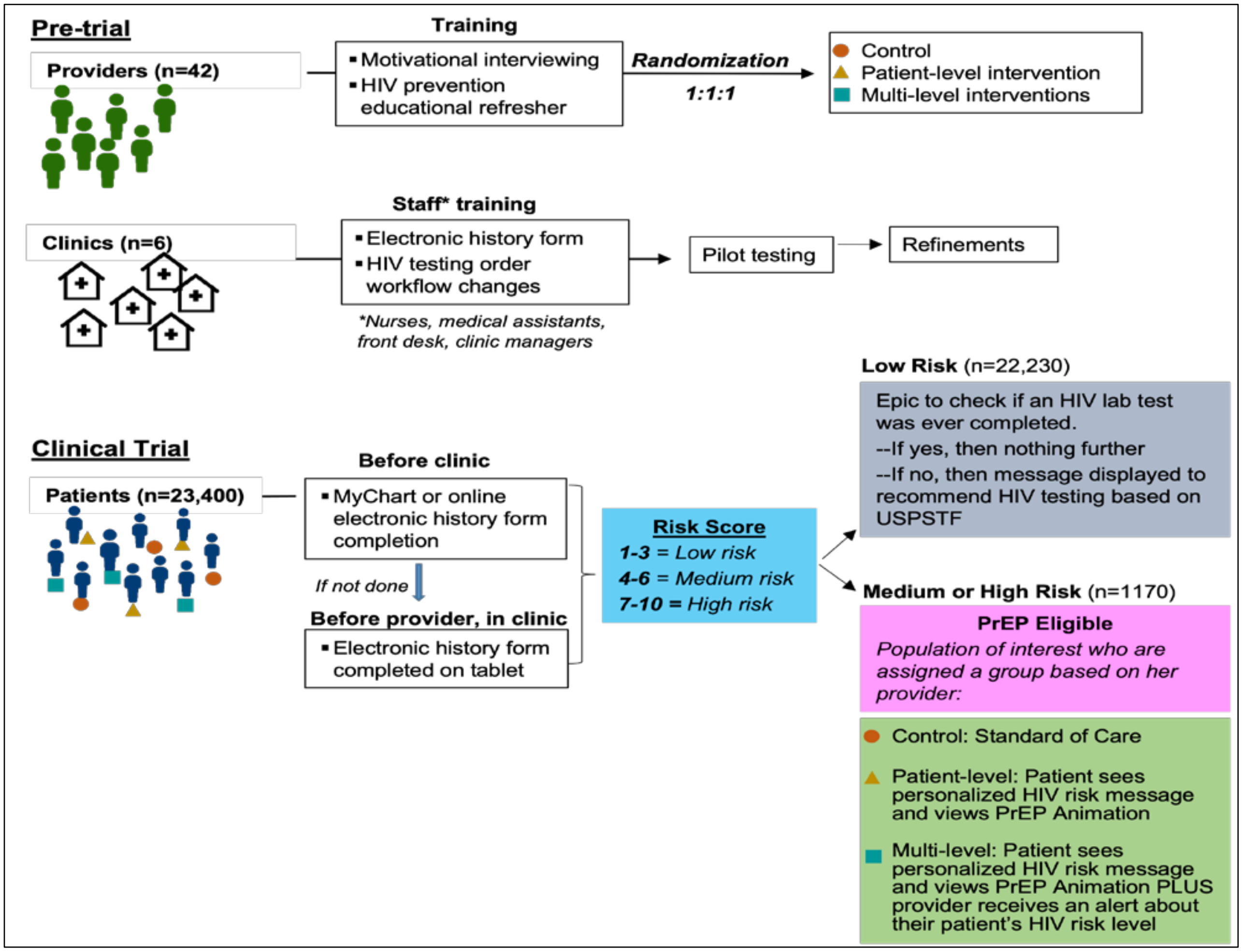

5. STUDY POPULATION

5.1 Selection of the Study Population

Provider eligibility: N=42 ObGyn providers will be randomized. We chose providers as the unit of randomization to minimize contamination of the arms as many providers see patients at more than one clinic site. This will reduce the provider from being exposed to both interventions and unconsciously applying them to patients in the control arm.

Patient eligibility: Cisgender women of the enrolled providers aged 15-65 years scheduled in Epic for one of the following visit types: well-woman exam (annual exam), STI testing, pre-conception counseling, or contraception consult (birth control) will complete the eSHxForm. Programming in Epic will assign each patient to a study arm based on their provider's assigned study group. The age range was selected based on the United States Preventive Services Task Force (USPSTF) HIV Testing Recommendations,[27] and PrEP FDA-approved age of 15 years.[4] Based on preliminary clinical data, we estimate that we will have 23,400 women who will complete the eSHxForm and an estimated 5% (n=1170) will fulfill eligibility for PrEP.

Staff eligibility: N= 50 clinical staff who work at one of the six Obgyn clinics. These will include front desk, medical assistants, nurses, and clinic managers.

5.2 Participant Inclusion Criteria

ObGyn providers
-Is a physician, nurse practitioner, midwife, or physician assistant
-Provide Obgyn care at Johns Hopkins Medicine ObGyn clinic or an affiliated FQHC (e.g., East Baltimore Medical Center, Baltimore Medical Services). These clinics include JHOC, Bayview BMO, Nelson 2, White Marsh, Remington, or EBMC.
-Employed by Johns Hopkins University or Johns Hopkins Hospital
-Willing and able to comply with the study procedures

Cisgender female patients
-Patients of enrolled providers aged 15-65 years
-Has an Epic visit type that includes one of the following: well-woman exam (annual exam), STI testing, pre-conception counseling, or contraception consult (birth control).

Staff
-Employed by Johns Hopkins University or Johns Hopkins Hospital
-Works at either JHOC, Bayview BMO, Nelson 2, White Marsh, Remington, or EBMC clinics.
-Is a front desk worker, medical assistant, nurse, or clinic manager.

5.3 Participant Exclusion Criteria

ObGyn providers
-Unable or unwilling to undergo motivational interviewing training
-Active plans to leave the institution
-Failure to maintain clinical privileges

Cisgender women
-Pregnant and has established prenatal care, i.e., a prenatal care visit
-Scheduled for a different visit type in Epic (e.g., Gyn problem visit for heavy bleeding)
-Non-English or non-Spanish speaking
-HIV-infected
-Unable or unwilling to complete the Electronic Sexual History Form (eSHxForm)

Staff
-Unwilling to participate in feedback sessions

6. STUDY PRODUCT/INTERVENTION(S)

6.1 Electronic Sexual History Form (eSHxForm):

Ideally, all patients would complete the eSHxForm before the clinic visit. Currently, patients are asked to complete a paper medical history form upon arrival to the clinics (JH Gyn New Patient Questionnaire 5/16, **Appendix A**). This form has detailed sexual history questions (e.g., type of sex [anal, vaginal, oral], sexual orientation, number of partners, number of new partners, prior STIs). We plan to transform the paper version into an electronic form. We will add a point value to each sexual history question and program Epic to summarize the score behind the scenes. Based on the score, different messages will be shown to the patient (**Appendix C**). The score will be visible in the EHR but it will not have a label assigned. In other words, there will not be any stigmatizing language (e.g., high HIV risk) attached to the score. The scoring algorithm is based on our other published research. [22,23] We will refer to this scored sexual health section as a Gyn HIV Risk Tool. Clinics will send the eSHxForm to patients to complete before the clinic visit. Messages will be sent via MyChart, email, or secure text messaging, per current clinical guidance. In the event that patients do not complete the eSHxForm before clinic, then they will receive a computer tablet upon arriving to their visit for completion while waiting for the provider.

Based on our experience with a different Gyn clinic-based project among our gyn cancer patients that used an electronic history form, approximately 65% of patients completed the pre-visit documentation. The remainder were asked to complete it on their own using clinic tablets while they waited for the provider. A small minority (<5%) were unable to complete the questionnaire and required the assistance of clinic staff. We will ask our medical assistants to assist the patient, but if she is unable to complete the eSHxForm, then the patient will not be included. Whether patients will respond differently to the eSHxForm, including the Gyn Risk Tool section depending on how it is administered or whether this will affect PrEP uptake is unknown. As part of our exploratory analysis, we will examine whether the mode of administration is related to responses to the Gyn HIV Risk Tool. In a sensitivity analysis for our primary outcome, we will also examine whether our results are robust to the administration mode, excluding those who used the less common modes.

6.2 Epic Best Practice Alerts (BPAs)

6.2.1 Medical assistant or other clinic staff BPA.

We will create a BPA that will be displayed in Epic only if patients who scored low on the eSHxForm desire an HIV test. The BPA will allow the medical assistant to notify the provider of the patient's desire for HIV testing and that an order has been pended in Epic for their signature, after routine HIV counseling (see **Appendix B** for example).

6.2.2 Obgyn Provider BPA.

As part of a previous PrEP project in the ObGyn clinics, we developed and implemented BPAs to assist providers with the identification of patients who may be PrEP eligible. The BPAs were displayed when a provider placed an antibiotic order PLUS the patient tested positive for a sexually transmitted infection. The alerts were well received by providers and led to increased PrEP discussions during its implementation. We will relaunch these BPAs as part of the 3rd Arm (multilevel intervention arm). The alert will include a description of the patient's HIV risk level based on how patients answer the sexual health questions) and a decision aid (ask if the provider would like to discuss PrEP and add the required laboratory tests before starting PrEP (see **Appendix B** for example).

6.3 PrEP videos

Our research group developed patient (video clip here) and provider (video clip here) animations about PrEP. The videos were informed by patients and providers, and peer-reviewed.[31] The Hopkins' pharmacy lead and patient education director reviewed the videos and approved them for clinical use. The videos are available in Epic under the Hopkins Program Library. The provider will view the provider animation during their training and the patient will be able to view the animation via a hyperlink embedded in the eSHxForm.

7. STUDY PROCEDURES

7.1 ObGyn Providers:

7.1.1 Recruitment will be via email, flyers (print and electronic), and word of mouth. Providers will not be recruited nor consented by a direct supervisor. Providers responding to an email or flyer will discuss the study with a research coordinator, who is not a direct supervisor. Recruitment will take place during providers meetings, before scheduled educational sessions, during scheduled work breaks, and other routine gatherings of providers.

7.1.2 Written informed consent will be obtained. Informed consent will be done by an interactive question and answer process where the potential subject will be asked specific questions to determine her understanding of the purpose, procedures, risks and benefits in participating in the study.

7.1.2 Motivational interview techniques will be presented as a Grand Rounds presentation for the entire department on September 29, 2022. Co-investigator from the University of Michigan will lead these efforts. Grand Rounds presentations are video recorded and can be viewed afterward. Also, brief (<25 minutes) online training modules will be created by our co-investigator for the participating ObGyn providers. The online module will test comprehension and basic skills (some sample questions will

include: identifying open-ended vs. closed-ended questions; understanding MI-consistent strategies; using rating scales to assess motivation, readiness, and confidence, etc.) We do not expect anyone to become an expert in motivational interviewing, however, the principles and basic skills can be taught briefly. Additionally, providers will be encouraged to view additional online modules quarterly. Immediate goals after viewing the training will be for providers to identify and understand the processes for implementing MI in their patient interactions (e.g., reflective statements), developing discrepancy, rolling with resistance, exploring ambivalence. Providers will learn how to avoid "MI traps" and how to implement communication strategies to help promote sexual health behavior change.

7.1.3 PrEP animations will be required to view. The 2 ½ minute animation will be sent to providers based on their preference. The animation provides brief educational material about PrEP.

7.1.4 Semi-structured in-depth interviews to evaluate the project (e.g., mainly processes) will occur throughout the project until we obtain feedback from 10-12 providers. We are not interested in the providers themselves for this qualitative portion and will not collect any of their PHI as part of these in-depth interviews.

7.2 Cisgender Female and Transgender Male Patients

7.2.1 All patients of providers who have the eligible visit types scheduled will be enrolled. We will request for them to complete the eSHxForm before arrival at the clinic or immediately before seeing a provider.

7.2.2 Waiver of informed consent is being requested (see below **Section 12.5.3**) for the eSHxForm and clinical trial procedures. Whereas a waiver of documentation of consent (e.g., oral consent) is being requested for in-depth interviews.

7.2.3 Semi-structured in-depth interviews to evaluate the project (e.g., mainly processes) will occur throughout the project until we obtain feedback from 10-12 patients from each HIV risk level (n=30-36, low, medium, and high). We are not interested in the patients themselves for this portion and will not collect any of their PHI as part of these in-depth interviews.

7.3 Clinic staff and other key stakeholders

7.3.1 Recruitment will be via email, flyers (print and electronic), and word of mouth. Clinic staff will not be recruited nor consented by a direct supervisor. Clinical staff responding to an email or flyer will discuss the study with a research coordinator, who is not a direct supervisor. Recruitment will take place during clinical staff meetings, before scheduled educational sessions, during scheduled work breaks, and other routine gatherings of clinical staff.

7.3.2 A waiver of documentation of consent is being requested for the focus group discussions and in-depth interviews.

7.3.3 Focus group discussions will be held at each clinic during regularly scheduled staff meetings to discuss the project, possible changes to the workflow, and address any concerns.

7.3.4 Semi-structured in-depth interviews to evaluate the project (e.g., mainly processes) will occur throughout the project until we obtain feedback from 10-12 clinic stakeholders. We are not interested in the staff members themselves and will not collect any of their PHI.

7.4 Fidelity checks

7.4.1 A research coordinator will observe the implementation of the study at the clinic and take field notes about the workflow, patients' questions, and providers' and stakeholders' concerns. The field notes will be analyzed every six months and used for cost analyses. Additionally, at every 6-month checks, the proportion of consecutive eligible patients who complete the eSHxForm will be assessed per clinic. If

completion is <75%, then research staff will obtain immediate feedback from clinical staff about barriers to completion and devise a plan to increase completion.

7.4.2 Focus group discussions for process evaluations (every 6 months for 18 months)
Additionally, we will conduct stakeholder virtual focus group discussions (FGD) during standing clinic staff meetings twice yearly. The FGDs will ask the following questions: **R** – How do you suggest we reach more patients?; **A** – Describe how well you felt equipped to deliver the interventions with the training you received? What would be necessary to build skills and confidence in intervention delivery in the future?; **I** – How do you report any suggestions you may have to improve the study procedures and delivery of the interventions?; **M** – What is the likelihood that you would continue with the interventions? What do you think would be needed for other staff to deliver the interventions in the future? We will not address **E**ffectiveness until study end as to avoid interfering with the clinical outcomes.

7.4.3 Findings from the field notes and FGDs will be adopted and guide the adjustment of our study, including optimizing workflow, improving training updates for providers, and addressing concerns from patients, providers, and other stakeholders.

8.0 STATISTICAL CONSIDERATIONS

We have rigorous methods to evaluate the interventions and implementation strategy. We will use the RE-AIM framework to guide the statistical plan (**Table 1**). JHU will be responsible for quantitative data analysis and University of Michigan will be responsible for qualitative data analysis.

Table 1. Intervention and Implementation Quantitative and Qualitative Outcomes using the RE-AIM Framework

	Intervention Outcomes	Implementation Strategy Outcomes	Level	Data Source
**REACH**	Number of patients with an eligible clinic visit who complete electronic screening assessments	Number of screening assessments that are completed electronically either via portal or in-clinic on tablet	Patient	Appointment scheduling log; EHR-programmed data transferred directly into RedCap database
	Number of patients who were excluded		Patient and Clinic	Appointment scheduling log; Observations, field notes
	Number of patients who view the animation		Patient	EHR-programmed data transferred directly into RedCap database
	QUALITATIVE: What strategies do you think worked best to capture eligible patients? What could the clinical staff and providers have done better to reach more people?			
**EFFECTIVENESS**	Number of patients who initiate PrEP (primary outcome)	Number of providers who document offering of PrEP	Patient and Providers	EHR chart review
		Number of PrEP prescriptions written	Providers	EHR discrete data element
	Number of patients who continue PrEP at 3-, 6-, 9-mos	Number of PrEP prescriptions written	Providers	EHR discrete data element
	Number of patients who are tested for HIV	Number of providers who document offering of HIV test	Patient and Providers	EHR lab data for HIV testing; EHR chart review
	QUALITATIVE: What surprised you about the outcomes of the training, counseling, clinical care and/or treatment you received? How would you like to see the data presented?			
**ADOPTION**		Number of clinics that use electronic screening assessments	Clinic	Appointment scheduling log; RedCap database; Stakeholder feedback
		Number of clinics that provide tablets to patients		
		Number of clinics that assist patients to complete electronic screening assessments		
	QUALITATIVE: What could the team have done better to reach and recruit more providers? What are your perceptions of the training that was offered?			
**IMPLEMENTATION**		Number of providers who decline the recommendations included in the alert	Provider	EHR-programmed data transferred directly into RedCap database
		Number of clinics that complete assessments in other EHR locations that are not a part of this project	Clinic	EHR chart review Observations, field notes Checklists
	QUALITATIVE: Do you think the interventions were delivered according to plan? What costs (including time and burden, not just money) need to be considered?			

**MAINTENANCE**	Above measures at 6 months after sample size achieved and research staff no longer providing support	Provider	EHR-programmed data transferred into RedCap database; chart review
		Clinic	
	QUALITATIVE: What is the likelihood that you would continue to support this intervention? What do you think would be needed for other women, providers, or staff to find this intervention meaningful?		

8.1 Primary endpoints

8.1.1 Intervention: Proportion of PrEP prescription 4 weeks after clinic visit

8.1.2 Implementation Strategy: Proportion of patients who complete the eSHxForm, number of providers declining automated orders (timeframe: q6 months)

8.2 Secondary endpoints

8.2.1 Intervention: Proportion of HIV test completion 4 weeks after clinic visit

8.2.2 Implementation Strategy: cost-effectiveness, return on investment (ROI), budget impact, number clinics that complete assessments as intended

8.3 Sample Size Estimate and Power.

A priori sample size calculation for the three-arm RCT was based on the expected annual patient volume across six clinics of approximately 7,800. We make the conservative assumption that 5-10% of these will score medium/high-risk on the Gyn HIV Risk Tool based on our preliminary studies,[32] yielding 390 patients annually, with a total of 1,170 over three years of enrollment. Our estimate is conservative as we found that close to 70% of patients in one of our clinics scored medium/high, but these were adolescents and young adult women who have the highest prevalence of STIs and risk behaviors.[33] These 1,170 patients will be seen by an expected 42 providers, where providers will be randomized to receive the potential intervention along with their patients. Given that patients using the same provider are not likely to be strongly related to each other in their choices for PrEP uptake (our co-primary outcome), we assume a low intra-provider correlation (IPC) of 0.01, with on average 28 patients per provider per year, yielding a design effect of 1 + (28 – 1)*IPC = 1.27. Given these assumptions, if we estimate PrEP uptake at 6% (prelim data), we will be able to detect a difference of 9% (from 6 to 15%) between arms with 80% power. We will examine the intervention effect within specific race/ethnicity groups. We will include race and ethnicity in our minimizing of differences between arms at randomization, so we can assume that race and ethnicity will be similarly distributed across arms. Assuming we have approximately 50% non-Latinx Black patient participants (as we do for the general patient population), we expect to enroll 585 non-Latinx Black patients, allowing for 80% power to detect a difference of 6% in PrEP uptake in the standard of care arm to 15% in the intervention arms, assuming the same design effect of 1.27.

8.4 Randomization and Blinding

ObGyn providers will be randomly assigned to one of three study arms based on minimization techniques developed by Pocock and Simon.[34] This minimization approach reduces covariate imbalances by utilizing non-uniform assignment probabilities for the different arms. The study arms will be balanced over the different clinics with equal sampling rates. Providers will be randomized in equal proportions between arms (14 providers per arm). All study personnel, study analysts, and patients will be masked to group assignment. Providers will be unmasked to group assignment. New providers who join a practice after another provider has left will undergo randomization.

8.5 Quantitative Analyses.

We will examine whether study randomization to the three intervention arms resulted in similar provider and patient groups by comparing patient and provider level demographics with descriptive statistics. Comparison of the primary outcome between arms will be conducted based on provider arm assignment, in accordance with intention to treat. We will use generalized linear mixed-effect models with logistic regression to examine the difference in PrEP uptake (primary outcome) across arms.[35] To account for the relationship among primary outcomes for patients with the same provider, we will specify a random provider effect. In addition, because some patients may return over the study period and be assisted by a different provider than their initial visit, we will specify a random effect for each patient, and assume a joint multivariate normal distribution for these provider and patient random effects.[36] We will adjust these comparisons for any demographic differences

observed between arms. We will examine the primary outcome stratified by race/ethnicity. In exploratory analyses, we will examine whether intervention effects attenuate over time, as providers and patients may become accustomed to the intervention. Secondary Outcomes: Similar to our methods for comparing the primary outcome between provider arms, we will also compare the rate of male partner HIV self-test completion (confirmed via text message or phone/video call) with generalized linear mixed-effect models with a provider and patient level random effects. We will use similar methods to compare the HIV test completion rates of male partner and patients who score low-risk. We will consider adjustment for factors that are imbalanced between arms in these analyses. In addition, we will also examine PrEP uptake among low-risk score patients to determine whether providers who are receiving the intervention are being influenced in their behavior more generally, or if the intervention effect is specific only to intervention patients. R (ver 4.1) will be used for analyses.

8.6 Other statistical considerations.

There is potential for significant interaction between interventions, determinants (for example, patient and provider characteristics), mechanisms (for example, motivation and skills), PrEP outcomes, and that these potential interactions likely have value for practice. It is possible that the intervention may be efficacious for some patients or providers, while not for others. In addition to our primary analysis, we will also examine who is most likely to use PrEP, since the determinants of PrEP uptake are not well-studied in this population, and whether the intervention was more effective for specific groups, focusing on differences by geographic area (zip code), patient marital status, patient insurance type, patient race and ethnicity, patient age, and patient risk score (medium vs high).

We expect differences in the rate of PrEP uptake for different providers that may depend on provider characteristics such as training or type of provider (e.g., nurse practitioner, physician), years in practice, and whether they are of the same race or gender as patients. We do not expect provider characteristics to impact trial outcomes because providers will be randomized to the intervention. However, we plan to examine the impact of provider characteristics on PrEP uptake independent of the intervention in secondary analysis, using generalized linear mixed-effect models, analogous to methods used in our primary analysis.

JHU will conduct a cost-effectiveness analysis (CEA) from the perspectives of a payer or an adopting organization (e.g., Medicaid), and using standard analytical methods recommended by the Second Panel on Cost-Effectiveness Analysis.[37,38] We will measure the relative efficiency of each active intervention (i.e., patient-focused intervention and patient-focused intervention plus EHR alerts) relative to standard of care in terms of their incremental cost-effectiveness ratios (ICER), calculated as the ratio of each intervention's incremental costs to its incremental benefits (e.g., incremental new STI/HIV test, new STI/HIV case diagnosed, number of new women on PrEP), and expressed in terms of dollars per health benefits. The ICER will be compared to a payer's "opportunity cost," i.e., willingness-to-pay (WTP) threshold to determine the intervention's acceptability in terms of its efficiency in generating health benefits, and under the particular implementation strategy. An ICER below the WTP will be indicative of good value for money produced by the intervention. Additionally, we will calculate the net monetary benefit and net health benefit of the intervention.

We will conduct a ROI by calculating the intervention's benefits, that is, the monetary values to the payer (or health care sector) of positive outcomes from the intervention (e.g., cost-offsets from avoided direct medical costs, as described above), minus its costs (i.e., incremental implementation costs, as measured above), expressed as a proportion of the incremental implementation cost.[38]

We will estimate the total budget needed to finance each intervention strategy. We will calculate the incremental costs of the intervention for all those in need in the population covered by the payer, on a "per capita" basis, per unit of time (1 year). The population in need will be estimated by multiplying the prevalence of the condition by the total number of individuals covered by the payer.

8.7 In-depth interviews

U of Michigan will implore an explanatory sequential design because the quantitative study is conducted first, followed by a qualitative evaluation to explain or expound on the quantitative findings.[39] This is reflected in the iterative process we will use for developing our PrEP education materials and the semi-structured IDI guide.

Certain quantitative measures (e.g., demographics, satisfaction rates) will be imported into the Dedoose™ database. Simple inferential statistics for two-way tables (e.g., Chi-Square) will be used to evaluate thematic differences between groups by site, demographics, and other key predictors identified in the quantitative analyses.

We will conduct 10-12 in-depth interviews (IDIs) for each of the following groups – patient, provider, and staff – for a total of 50-60 IDIs, which will allow for saturation of responses.[40,41]

9. ASSESSMENT OF SAFETY

9.1 Adverse Events (AE)

An adverse event is defined as any untoward medical occurrence in a clinical research participant administered an investigational product and which does not necessarily have a causal relationship with the investigational product. We are not administering an investigational product. However, any adverse events will be graded by the following scale:

- **Mild**: subject was aware of the adverse event, but she/he was still able to do all activities; no or minimal medical intervention/therapy required.
- **Moderate**: the subject had to discontinue some activities due to the adverse event; no or minimal medical intervention/therapy required.
- **Severe:** the subject was incapacitated by the adverse event and unable to perform normal activities; significant medical intervention/therapy required, hospitalization possible.

The investigator will then determine if an AE is related or unrelated to the subject's participation the study. The investigator or designee will assess the relationship of all AEs to the study product based on his/her clinical judgment.

- **Related** – The AE is known to occur with the study procedures, there is a reasonable possibility that the study procedures caused the AE, or there is a temporal relationship between the study procedures and the event. Reasonable possibility means that there is evidence to suggest a causal relationship between the study procedures and the AE.
- **Not Related** – There is not a reasonable possibility that the study procedures caused the event, there is no temporal relationship between the study procedures and event onset, or an alternate etiology has been established.

9.2 Expedited Adverse Event Reporting Requirements

Participants will be instructed to contact the study clinician to report not only danger signs, but also any events they may experience, except for life-threatening events, for which they will be instructed to seek immediate emergency care. Depending on the severity of the event, the clinician will instruct the participant to present to the study site (for more mild events) or to an emergency room (for more serious events) for immediate evaluation. Where feasible and medically appropriate, participants will be encouraged to seek medical care where the study clinician is based, and to request that the clinician be contacted upon their arrival, if possible.

Study site staff at JHU and U of Michigan will be informed that all AEs reported by or observed in enrolled study participants regardless of severity and presumed relationship to the study procedures must be documented. U of Michigan site staff will promptly report AEs to the JHU site PI (within 5 working days from the event). JHU site staff will report information on all AEs to the IRB promptly (within 10 working days of the event), as required by all applicable regulations and local IRB requirements. Additionally, information on all AEs will be included in reports to applicable government and regulatory authorities.

AEs must be reported on an expedited basis during the entire study duration for an individual subject (from study enrollment until study completion or discontinuation of the subject from study participation for any reason).

9.3 Serious Adverse Event
A serious adverse event (SAE) will be defined per U.S. Code of Federal Regulations (CFR) 312.32 and International Conference on Harmonisation (ICH), “Good Clinical Practice: Consolidated Guidance” (E6) and “Clinical Safety Data Management: Definitions and Standards for Expedited Reporting” (E2A), as AE occurring at any dose that:

- Results in death
- Is life-threatening
- Results in persistent or significant disability/incapacity
- Is a congenital anomaly/birth defect
- Requires inpatient hospitalization or prolongation of existing hospitalization

9.4 Protocol Deviations
Protocol deviations will be reported to JHU IRB per policy. We do not anticipate having any emergency or major protocol deviations as the study is minimal risk. Minor or administrative protocol deviations will be reported to the IRB at the continuing review. These include deviations which do not “affect the scientific soundness of the research plan or the rights, safety, or welfare of human subjects.”

10. CLINICAL MANAGEMENT

10.1 Management of Positive HIV Tests
Our implementing partner JHHWHP has extensive experience with HIV testing and linkage to care. We will offer their assistance to providers with patients who test positive as part of routine clinical care.

10.1.1. In routine care, a patient who tests HIV positive is advised that she should inform all sexual and needle-sharing partners of her positive HIV status or make arrangements for them to be informed by a third party.

10.1.2 The patient is offered assistance in notifying sexual partners. If the patient requests assistance, our implementing partner JHHWHP usually is notified.

10.1.3 If the patient is informed about her positive test result and her responsibility to inform sexual and needle-sharing partners and refuses to do so, our implementing partner JHHWHP will notify the provider responsible for ordering the HIV test about the patient's refusal to notify his/her sexual and needle-sharing partners in accordance with Code of Maryland Regulations, COMAR 10.18.04.03.

10.1.4 The patient will be notified that the Health Department will be notified of the confirmed positive test result.

10.1.5 The patient immediately will be linked to HIV care by our implementing partner JHHWHP as is currently done for clinical care.

10.2 Criteria for Early Termination of Study Participation
Participants may voluntarily withdraw from the study for any reason at any time. The PI may withdraw participants before their scheduled termination visit in order to protect their safety, and/or if participants are unable or unwilling to comply with study procedures.

10.3 Pregnancy
Women who are diagnosed with a early pregnancy during a preventative health visit will be included. Only those women who have initiated prenatal care will be excluded.

11. Data and Safety Monitoring

11.1 Sources of Materials

The research material is mostly clinical data collected from the EHR and stored into our REDCap database. Some data may require a manual chart review. All data will be presented anonymously, and only the study team members, the IRB, and other agencies allowed by law will have access to study binders. Material or data will be obtained specifically for research purposes.

Based on other similar studies conducted by the investigators, our study is of minimal risk and, therefore, we do not plan to have a formal Data Safety Monitoring Board. The PI will have sole responsibility for monitoring and oversight of problem/events. Dr. Coleman has completed several clinical studies, including randomized clinical studies, with a focus on STI testing and treatment, and HIV prevention, involving minors who are able to consent for themselves. Our states' laws permit an adolescent under 18 years old to consent to minimal risk research (such as this project) when receiving reproductive and sexual healthcare.

12. DATA HANDLING AND RECORDKEEPING

Study staff will create case report forms in RedCap with scheduled data pulls from Epic. We anticipate having monthly data pulls from Epic. Site Standard Operating Procedures will also be created as part of the study activation process. All case report forms that will be used as source documents will be identified.

There will be close coordination between protocol team members to track study progress, respond to queries about proper study implementation, and address other issues in a timely manner. After any protocol or consent changes, an email will be sent to both site investigators to inform about the most current version.

12.1 Investigator's Records

The study PI will maintain, and store in a secure manner, complete, accurate and current study records throughout the study. Study records include complete administrative documents, such as registration documents and reports related to the study, correspondence, informed consent forms, source documents, and records of all contacts with study participants. Study records will be retained on site for at least three years.

12.2 Data Integrity

Implementation of the clinical trial will be studied alongside effectiveness of the interventions. Every 6 months, the research team will review and analyze outcome data and processes via focus group discussions with clinic staff, field notes, and RedCap data analyses. Focus group discussions will occur during routinely scheduled staff meetings to identify barriers and facilitators to the research (see Table 1 above for RE-AIM framework to guide the discussions). Field notes will be taken by a research coordinator/assistant who will observe the implementation of the study at the clinic and take notes about the workflow, patients' questions, and providers' and stakeholders' concerns. Additionally, the proportion of consecutive eligible patients who complete the eSHxForm will be assessed per clinic. If completion is <75%, then research staff will obtain immediate feedback from clinical staff about barriers to completion and devise a plan to increase completion.

Focus group discussions and individual in-depth interviews will be facilitated by a JHU research staff member. JHU research staff will host the virtual focus group or interview using the videoconferencing software Zoom between U of Michigan research staff and JHU research participant(s). JHU will audio record the sessions. Participants will have the option to turn-off the video component of Zoom to minimize sharing of facial images. The resulting audio files will be saved on the SAFE Desktop. Only the JHU PI and designated research staff will have access to the SAFE Desktop audio files folder. The audio recordings from the SAFE Desktop will then be uploaded onto the Hopkins' approved transcription vendor's (GMR Transcription) platform. Once the audio has been transcribed verbatim, transcripts will be stored on the SAFE Desktop. The transcripts will be stripped of personal health identifiers (e.g., name), specific professional positions (e.g., division director, medical director), and clinic names. Focus group and interview guides do not ask a participant for identifying information since the qualitative research procedures are focused on the new clinical process and not the participants' medical information. After removing identifiers, JHU researchers will note where a pseudonym was inserted through use of [brackets] and a master log of all replacements, aggregations, or removals made will be created and kept in a separate file on SAFE Desktop. Once de-identified, the transcripts will be uploaded to OneDrive so research members from our collaborating academic institution (U of Michigan) are able to access the de-identified transcripts. U of Michigan researchers will use a cloud-based qualitative

software called Dedoose™ to help code and analyze the data. U of Michigan researchers will not download transcripts onto personal computers/laptops. Once the analysis has occurred, the findings will be shared to incorporate them into the trial.

13. HUMAN SUBJECTS PROTECTIONS

13.1 Potential Risks

13.1.1 Loss of Confidentiality

Although the study site will make every effort to protect the privacy and confidentiality of all study participants, it is possible that participants' involvement in the study could become known to others.

Given the sensitive nature of the proposed studies, the investigational team will keep the study information private to the extent possible by law. Where possible, clinical information will be identified by a unique number assigned to each individual research participant. Access to study records will be limited to the study team, including the Office of Human Research Protections, and Johns Hopkins University officials, where applicable and required by law. Enrolled patients will be assigned study numbers for data collection and evaluation. Publications in medical journals arising from this study will not include any names or other identities of the subjects involved.

The key linking research subjects to study numbers will be kept as an electronic file. Electronic files will be maintained in RedCap software on a JHU secure computer server. Access to the files will require a password provided to designated study investigators. Any paper files will be maintained in a locked cabinet located in the Division of Gynecologic Specialties with access limited to study investigators.

13.1.2 Genetic Testing

None

13.1.3 Embarrassment

Participants may feel uncomfortable or embarrassed answering questions about sensitive subjects (i.e., their sexual behaviors). The likelihood of emotional distress is low. Our ObGyn providers routinely collect sexual behavior information and are experienced in sexual history taking. Almost all of the eSHxForm questions are currently being asked as part of routine clinical care. Complaints of embarrassment or distress are very rare (<1%). Mild emotional distress is not considered a serious risk to the subject.

13.1.4 HIV Testing

Researchers are not ordering any HIV tests; the research team is interested in the HIV test placement and the results. The research will facilitate the decision making around placing the order in the EHR, but the provider has to consent the patient as per usual care and sign the laboratory order. In general, consent for HIV testing is documented in the patient’s medical record, as it is currently done as part of standard of care. Documentation in the medical record will state that the individual tested received pre and post-test counseling and was informed she/he had the right to refuse HIV testing without penalty. Patients who are 15 years of age and older will be included; therefore children may be potentially enrolled in this study. Under current laws in Maryland, minors are allowed to consent for HIV testing and treatment and other sexually transmitted infections without parental consent. To be consistent with these laws, patients who are under the age of 18 years of age and agree to PrEP or HIV testing during this study will be able to do so without parental consent as standard of care. Based on Maryland State section code §20-102 (c3), a minor has the same capacity as an adult to consent to: (1) treatment for or advice about drug abuse; (2) treatment for or advice about alcoholism; (3) treatment for or advice about venereal disease.

13.1.5 Social Harms

Social harms are any untoward social occurrences that happen to a participant as a result of their participation in the study. Examples include loss of employment, harassment by neighbors, shunned by family, rejection by partner, etc. Although social harms due to this study are expected to be negligible, they will be monitored closely throughout the study. Information on social harms will be actively solicited from participants and

recorded on case report forms and captured in the study database. Participants will also be encouraged to report any social harm on an ad hoc basis when it occurs before the study visits. In the event that a participant reports social harm, every effort will be made by study staff to provide appropriate care and counseling to the participant, and/or referral to appropriate resources for the safety of the participant as needed. Social harms that are judged by the Investigator of Record to be serious or unexpected will be reported to the IRB at least annually. The nature and frequency of these social impact reports will be monitored by the protocol team on a regular basis.

13.1.6 Psychological risks: The length of the clinic visit may be lengthened if the patient would like to discuss HIV and PrEP. Providers may feel stress. The research is not designed to increase the visit length and the goal is to facilitate discussions that usually happen during these types of office visits.

13.2 Protections Against Risk

Protecting Privacy. Good Clinical Practice guidelines will be in place to maintain participants' confidentiality with regard to health status during clinical investigations, and medical records documentation. Data collection forms will include study numbers only. The code sheet with subjects' identification data (such as names and medical record ID numbers) and study numbers will be filed in RedCap with access limited to designated study staff. To ensure security of computer-stored information, research data will be stored by identification number in a separate RedCap datafile. Any published results from the study will be in the form of tabular descriptions of groups only. These processes have proved effective in multiple prior research projects.

Procedures for Minimizing risk. Study investigators will closely monitor for adverse events. Our team operates under Good Clinical Practice guidelines. The principal investigator will ascertain that all co-investigators are current with their IRB certification for participation in human subjects research.

13.4 Benefits

Participants in the study may or may not benefit from being in this study. Providers might obtain motivational interviewing skills and additional education about PrEP. Patients might gain a better understanding of their personal HIV risk and education about HIV prevention methods.

The risks to individual participants are outweighed by potential benefits to the scientific community and to society. The risks of the study are extremely small; we have built an adequate protection against these potential risks.

13.5 Informed Consent

13.5.1 Providers

Written informed consent will be obtained from all providers by a Hopkins' study investigator or nurse, usually an experienced research coordinator trained for this purpose and approved to consent research subjects by the IRB. Only Hopkins' study team members will obtain written informed consent. Study team members from the University of Michigan will not obtain written informed consent. A direct supervisor will not consent providers. The research coordinator will obtain written informed consent using all the elements of informed consent (21 CFR 50.25 and ICH GCP 4.8.10). Research participants will be given ample time to review the informed consent. HIPAA procedures will be followed and properly documented. To document informed consent procedures, all signed original informed consent forms (ICF) are filed in each research participant's source document file with source notes denoting the date and time of consent(s), the person(s) involved with the consent process (e.g., study nurse or coordinator), and a statement that the research participant was indeed given a copy of the signed and dated ICF to take home. Providers will be consented to participate in motivational interviewing, viewing PrEP animations, the clinical trial, and in-depth interviews.

13.5.2 Patients

13.5.2.1 Waiver of Informed Consent

We are requesting a waiver of consent for patients who are cisgender women or transgender men who will complete the eSHxForm (aka Gyn Risk Tool) and included as part of the evaluation of the effectiveness trial. This waiver request is based on the following in accordance with 45_CFR_46.116(d)(1-4):

(1) The study is minimal risk because the probability and magnitude of harm or discomfort anticipated in the research are not greater in and of themselves than those ordinarily encountered in daily life or during the performance of routine physical or psychological examinations or tests.

The main risks include breach of confidentiality and possible risks of change from standard of care (e.g., providing an HIV risk messaging). However, the questions that are scored are routine components of a comprehensive sexual history that is recommended by national organizations (e.g., CDC and American College of Obstetricians and Gynecologists (ACOG)). The most significant risk to patients will be loss of confidentiality, which is viewed as minimal. All of the methods utilized in this study are currently accepted, recommended and utilized methods for routine clinic visits in different departments and other EHR studies. Furthermore, there is minimal difference in risk between the interventions. We will display a recommendation to undergo HIV testing to patients who score low-risk, which is a national recommendation by USPSTF. The patient has the opportunity to decline and the provider will discuss the HIV test order. While some patients will receive additional educational information about PrEP and others will have a provider who had received a real-time Epic alert about PrEP, these do not increase the risk above the standard of care. Currently, some providers are adept at discussing PrEP, whereas others may forget or miss opportunities to discuss it with their patients. Patients who access PrEP or HIV testing services in routine health care settings are exposed to this same risk. Therefore, the risks involved in this study are no more than what the patient would experience in standard care.

The Principal Investigator will assume full responsibility for the protection of all study-related documents and datasets, including those that contain protected health information. The Research Coordinator and Principal Investigator will oversee all data collection. All electronic data will be held in an electronic database (Redcap). The database is password protected and stored on a Hopkins' secure network. This network includes firewall protection and the data can only be accessed by designated members of the study team. Any paper documents will be stored in a locked file cabinet in the Research office. These data will be manually entered into the main electronic database. During this project, all data will be kept in secure locations either in the office of the Principal Investigator or the office of the Research Coordinator.

(2) It does not violate patients' rights. The research will provide clinical decision aids about PrEP and HIV testing by asking standard-of-care questions, but the patient will still be able to decide whether either occurs. The risks involved in this study are no more than what the patient would experience in standard care and each patient will be able to decide whether to accept PrEP or HIV testing. Additionally, we will have two disclaimers in the eSHxForm. The first will be listed before the sexual history questions that are currently included on the paper history form. The purpose of this first disclaimer is to inform patients that their responses to the questions may prompt additional messaging. It will state, *"Based on your answers to the following questions, you may be shown additional information about your sexual health. Also, your Obgyn provider might receive this information and discuss your sexual health during the visit."* The second disclaimer will be listed before the sexual history questions that are not routinely asked by all of our providers. However, these questions are asked during a routine prenatal care visit using the Epic Risk Assessment Flowsheet, and we would like to include them for non-pregnant patients as well. It will state, *"We ask some of our patients the following questions but have not routinely asked all of our patients. Therefore, the answer to these questions will be used for research to help us determine if we should begin to ask ALL patients these questions in the future. You can choose to skip these questions."*

(3) Separate written informed consent cannot practicably be carried out without a waiver because of the large number of consecutive patients included in the six clinical sites, and the potential for biased participation in the study. There is an abundance of literature about the lack of HIV risk perception on part of the patient and provider.[7,12,15,16] Patients will not participate because they may believe they are not at-risk for HIV, which essentially was the impetus to design this study. It is essential that we have close to 100% participation in this study in order to assess how we can assist patients and providers with discussion about

HIV prevention. We believe this waiver of consent will not adversely affect the rights and welfare of the subjects involved in this project, as they will only be subjected to current standards of care and they will all have the right to refuse PrEP and HIV testing.

(4) Each patient will have an experience that does not differ significantly from usual care. Our design simply standardizes practices between providers.

13.5.2.2 Waiver of Documentation of Informed Consent

Patients will be asked to participate in in-depth interviews, which are likely to occur via telephone or a secure HIPAA-compliant video platform. We will not collect any PHI about them and we are only interested in process measures about the intervention. Based on the availability of the research staff, we will purposively sample patients in each of the six clinics until we reach our sample size (n=10-12). These participants will undergo an oral consent process.

Oral informed consent will be obtained from all participants by a Hopkins' study investigator or nurse, usually an experienced research coordinator trained for this purpose and approved to consent research subjects by the IRB. The University of Michigan will not participate in the consent process. The research coordinator will obtain informed consent using all the elements of informed consent (21 CFR 50.25 and ICH GCP 4.8.10). Research participants will be given ample time to review the informed consent form beforehand. HIPAA procedures will be followed and properly documented. To document informed consent procedures, the oral consent forms are filed in each research participant's source document file with source notes denoting the date and time of consent(s), the person(s) involved with the consent process (e.g., study nurse or coordinator), and a statement that the research participant was indeed given a copy to take home.

13.5.2.3 Non-English Speaking Patients

Potential research participants who do not speak English will not be automatically excluded. The proposing investigators strongly encourage the conduct of human subjects research to allow full participation of non-English speakers. To enable the enrollment of non-English speakers and to be fully compliant with the Department of Health and Human Services (DHHS) regulations (45 CFR 46.116 and 45 CFR 46.117) and FDA regulations (21 CFR 50.25 and 21 CFR 50.27) participants who do not speak English must be provided with both a written consent document in a language understandable to them and a translator fluent in both English and the participant's spoken language. The consent process may also be completed using an interpreter in the preferred language by phone. Interpreter services are readily available across our participating health systems.

13.5.3 Clinic Staff

13.5.3.1 Waiver of Documentation of Informed Consent

Clinic staff will be asked to participate in focus groups and in-depth interviews, which are likely to occur via telephone, a secure HIPAA-compliant video platform, or in-person. We will not collect any PHI about them and we are only interested in process measures about the intervention. Based on the availability of the research staff, we will purposively sample clinic staff in each of the six clinics until we reach our sample size (n=10-12). These staff members will undergo an oral consent process.

Oral informed consent will be obtained from all participants by a Hopkins' study investigator or nurse, usually an experienced research coordinator trained for this purpose and approved to consent research subjects by the IRB. A direct supervisor will not consent clinic staff. The University of Michigan will not participate in the consent process. The research coordinator will obtain informed consent using all the elements of informed consent (21 CFR 50.25 and ICH GCP 4.8.10). Research participants will be given ample time to review the informed consent form beforehand. HIPAA procedures will be followed and properly documented. To document informed consent procedures, the oral consent forms are filed in each research participant's source document file with source notes denoting the date and time of consent(s), the person(s) involved with the consent process (e.g., study nurse or coordinator), and a statement that the research participant was indeed given a copy to take home.

13.6 Importance of the Knowledge to be Gained

This line of research will identify the most effective intervention and implementation strategy that will promote HIV testing and PrEP initiation among reproductive-age cisgender women and transgender men in a region with a high HIV prevalence. We expect that our findings will be generalizable to other Obgyn and primary care settings. We believe the small risks to which healthy volunteers will be exposed in this study are well-balanced by the benefits of advancing our knowledge.

13.7 Compensation

As part of recruiting and retention efforts, subjects will be compensated monetarily for their time, inconvenience, and participation in the qualitative parts of study. Patients will receive $75, and providers and clinical staff will receive $75 for their participation in the 45-minute in-depth interviews that will identify barriers and facilitators of effective implementation of our multi-level and multi-component intervention. Focus group participants will be entered into a lottery for a chance to win $50. Each time a participant participates in a focus group session, they will receive a lottery ticket to enter the drawing and increase their chances of winning. There will be one lottery per clinic site per year.

13.8 Costs

There will be no costs to the subjects.

14. LABORATORY EVALUATIONS

14.1 Laboratory Evaluations and Specimen Collection

None. All laboratory tests will be ordered and signed by providers and not the research team.

14.2 Specimen Storage and Future Use

None

14.3 Biohazard Containment

N/A

REFERENCES

1. Women CoGPaCoHCfU. Health Care for Transgender and Gender Diverse Individuals. In: American College of Obstetricians and G, ed. Committee Opinion2021.

2. Centers for Disease Control and Prevention. Estimated HIV Incidence and Prevalence in the United States, 2014-2018. HIV Surveillance Supplemental Report 2020. 2020.

3. Services USDoHaH. HIV National Strategic Plan for the United States: A Roadmap to End the Epidemic 2021-2025. 2021.

4. Administration USFaD. Truvada Prescribing Information.

5. Bailey JL, Molino ST, Vega AD, Badowski M. A Review of HIV Pre-Exposure Prophylaxis: The Female Perspective. Infect Dis Ther 2017;6(3):363-382. DOI: 10.1007/s40121-017-0159-9.

6. Calabrese SK, Underhill K, Earnshaw VA, et al. Framing HIV Pre-Exposure Prophylaxis (PrEP) for the General Public: How Inclusive Messaging May Prevent Prejudice from Diminishing Public Support. AIDS Behav 2016;20(7):1499-513. DOI: 10.1007/s10461-016-1318-9.

7. Carley T, Siewert E, Naresh A. Interest in Pre-exposure Prophylaxis (PrEP) for HIV is Limited Among Women in General Obstetrics and Gynecology Setting. AIDS Behavior 2019;23:2741-2748. DOI: 10.1007/s10461-019-02529-1.

8. Huang YA, Zhu W, Smith DK, Harris N, Hoover KW. HIV Preexposure Prophylaxis, by Race and Ethnicity - United States, 2014-2016. MMWR Morb Mortal Wkly Rep 2018;67(41):1147-1150. DOI: 10.15585/mmwr.mm6741a3.

9. Karletsos D, Stoecker C. Impact of Medicaid Expansion on PrEP Utilization in the US: 2012-2018. AIDS Behavior 2021;25:1103-1111. DOI: 10.1007/s10461-020-03070-2.

10. Raifman JR, Schwartz SR, Sosnowy CD, et al. Brief Report: Pre-exposure Prophylaxis Awareness and Use Among Cisgender Women at Sexually Transmitted Disease Clinic. J Acquir Immune Defic Syndr 2019;80:36-39. DOI: 10.1097/qa0000000000001879.

11. Sheth AN, Rolle CP, Gandhi M. HIV pre-exposure prophylaxis for women. J Virus Erad 2016;2:149-55.

12. Scott RK, Hull SJ, Richards RC, Klemmer K, Salmoran F, Huang JC. Awareness, acceptability, and intention to initiate HIV pre-exposure prophylaxis among pregnant women. AIDS Care 2021;19:1-13. DOI: 10.1080/09540121.196870.

13. Tabacco L, Chung S-e, Perin J, Huettner S, Butz A, Trent M. Relationship Status and Sexual Behaviors in Post-Pelvic Inflammatory Disease (PID) Affected Urban Young Women: A Sub-Study of a Randomized Controlled Trial. Nursing and Health Care 2018;4. DOI: 10.23937/2469-5823/1510088.

14. Hong JN, Farel CE, Rahangdale L. Pharmacologic prevention of human immunodeficiency virus in women: practical approaches for the obstetrician and gynecologist. Obstet Gynecol Surv 2015;70(4):284-90. DOI: 10.1097/ogx.0000000000000170.

15. Krakower D, Mayer KH. Engaging healthcare providers to implement HIV pre-exposure prophylaxis. Curr Opin HIV AIDS 2012;7(6):593-9. DOI: 10.1097/COH.0b013e3283590446.

16. Seidman D, Carlson K, Weber S, Witt J, Kelly PJ. United States family planning providers' knowledge of and attitudes towards preexposure prophylaxis for HIV prevention: a national survey. Contraception 2016;93(5):463-9. DOI: 10.1016/j.contraception.2015.12.018.

17. Pringle K, Merchant RC, Clark MA. Is self perceived HIV risk congruent with reported HIV risk among traditionally lower HIV risk and prevalence adult emergency department patients? Implications for HIV testing. AIDS Patient Care STDS 2013;27:573-84. DOI: 10.1089/apc.2013.0013.

18. Adekeye OA, Abara WE, Xu J, Lee JM, Rust G, Satcher D. HIV Screening Rates among Medicaid Enrollees Diagnosed with Other Sexually Transmitted Infections. PLoS One 2016;11(8):e0161560. DOI: 10.1371/journal.pone.0161560.

19. Liao C, Golden WC, Anderson JR, Coleman JS. Missed Opportunities for Repeat HIV Testing in Pregnancy: Implications for Elimination of Mother-to-Child Transmission in the United States. AIDS Patient Care STDS 2017;31(1):20-26. (In eng). DOI: 10.1089/apc.2016.0204.

20. Koren DE, Nichols JS, Simoncini GM. HIV Pre-Exposure Prophylaxis and Women: Survey of the Knowledge, Attitudes, and Beliefs in an Urban Obstetrics/Gynecology Clinic. AIDS Patient Care STDs 2018;32:490-494. DOI: 10.1089/apc.2018.0030.

21. Ward H, Ronn M. Contribution of sexually transmitted infections to the sexual transmission of HIV. Curr Opin HIV AIDS 2010;5(4):305-10. DOI: 10.1097/COH.0b013e32833a8844.

22. Ronda J, Gaydos CA, Perin J, Tabacco L, Coleman JS, Trent M. Does the Sex Risk Quiz Predict Mycoplasma genitalium Infection in Urban Adolescents and Young Adult Women? Sex Transm Dis 2018;45(11):728-734. DOI: 10.1097/olq.0000000000000874.

23. Trent M, Coleman JS, Hardick J, et al. Clinical and sexual risk correlates of Mycoplasma genitalium in urban pregnant and non-pregnant young women: cross-sectional outcomes using the baseline data from the Women's BioHealth Study. Sex Transm Infect 2018;94(6):411-413. DOI: 10.1136/sextrans-2017-053367.

24. Centers for Disease Control and Prevention. US Public Health Service: Preexposure prophylaxis for the prevention of HIV infection in the United States—2021 Update: a clinical practice guideline. 2021.

25. Auerbach JD, Kinsky S, Brown G, Charles V. Knowledge, attitudes, and likelihood of pre-exposure prophylaxis (PrEP) use among US women at risk of acquiring HIV. AIDS Patient Care STDS 2015;29:102-10. DOI: 10.1089/apc.2014.0142.

26. Hirschhorn L, Brown R, Friedman E, et al. Black Cisgender Women's PrEP Knowledge, Attitudes, Preferences, and Experience in Chicago. J Acquir Immune Defic Syndr 2020;84:497-507. DOI: 10.1097/QAI.0000000000002377.

27. Force USPST, Owens DK, Davidson KW, et al. Screening for HIV Infection: US Preventive Services Task Force Recommendation Statement. JAMA 2019;321(23):2326-2336. DOI: 10.1001/jama.2019.6587.

28. Mayer KH, Agwu A, Malebranche D. Barriers to the Wider Use of Pre-exposure Prophylaxis in the United States: A Narrative Review. Adv Ther 2020;37(5):1778-1811. (In eng). DOI: 10.1007/s12325-020-01295-0.

29. Henny KD, Duke CC, Geter A, et al. HIV-Related Training and Correlates of Knowledge, HIV Screening and Prescribing of nPEP and PrEP Among Primary Care Providers in Southeast United States, 2017. AIDS Behav 2019;23(11):2926-2935. (In eng). DOI: 10.1007/s10461-019-02545-1.

30. Liu A, Cohen S, Follansbee S, et al. Early experiences implementing pre-exposure prophylaxis (PrEP) for HIV prevention in San Francisco. PLoS Med 2014;11(3):e1001613. (In eng). DOI: 10.1371/journal.pmed.1001613.

31. Young A, Okonkwo O, Gingher E, Coleman J. Developing Educational Animations on HIV PrEP

for Women: A Qualitative Study. JMIR Formative Research 2022;PAP.

32. Fruhauf T, Coleman J. A Missed Opportunity for U.S. Perinatal Human Immunodeficiency Virus Elimination Pre-exposure Prophylaxis During Pregnancy. Obstetrics and Gynecology 2017;130:703-709. DOI: 10.1097/aog.0000000000002258.

33. Ronda J, Gaydos CA, Perin J, Tabacco L, Coleman J, Trent M. Does the Sex Risk Quiz Predict Mycoplasma genitalium Infection in Urban Adolescents and Young Adult Women? Sex Transm Dis 2018;45:728-734. DOI: 10.1097/olq.0000000000000874.

34. Pocock SJ, Simon R. Sequential treatment assignment with balancing for prognostic factors in the controlled clinical trial. Biometrics 1975;31:103-15.

35. Rabe-Hasketh S, Skrondal A, Pickles A. Maximum likelihood estimation of limited and discrete dependent variable models with nested random effects. Journal of Econometrics 2005;128:301-323.

36. Barr DJ, Levy R, Scheepers C. Random effects structure for confirmatory hypothesis testing: Keep it maximal. Tily, Harry J 2013;68. DOI: 10.1016/j.jml.2012.11.001.

37. Drummond MF, Sculpher MJ, Claxton K, Stoddart GL, Torrance G. Methods for the Economic Evaluation of Health Care Programmes. 2015.

38. Neumann PJ, Sanders GD, Russel L, Siegel JE, Ganiats TG. Cost-Effectiveness in Health and Medicine. 2016.

39. Busetto L, Wick W, Gumbinger C. How to use and assess qualitative research methods. Neurol Res Pract 2020;2:14. (In eng). DOI: 10.1186/s42466-020-00059-z.

40. Guest G, Bunce A, Johnson L. How Many Interviews Are Enough?: An Experiment with Data Saturation and Variability. Field Methods 2006;18:59-82.

41. Guest G, Namey E, Chen M. A simple method to assess and report thematic saturation in qualitative research. PLoS One 2020;15(5):e0232076. (In eng). DOI: 10.1371/journal.pone.0232076.
