# Supplementary material for: An effectiveness-implementation trial protocol to evaluate PrEP initiation among U.S. cisgender women using eHealth tools vs. standard care
Source: Front Reprod Health. 2023 Jun 8;5:1196392. doi: 10.3389/frph.2023.1196392 (PMC10285440; doi:10.3389/frph.2023.1196392)
Supplement: Supplementary file 4 [file Datasheet4.pdf]

JOHNS HOPKINS MEDICINE

Office of Human Subjects Research
*Institutional Review Boards*

1620 McElderry Street, Reed Hall, Suite B-130
Baltimore, Maryland 21205-1911
410-955-3008
410-955-4367 Fax
e-mail: jhmeirb@jhmi.edu

**Date:** August 17, 2022

## APPLICATION APPROVAL

| | |
|---|---|
| **Review Type:** | Expedited |
| **Principal Investigator:** | Jenell Coleman |
| **Number:** | IRB00331706 |
| **Title:** | Clinic-based HIV Identification and Prevention Project using Electronic Resources (CHIPPER) |
| **Committee Chair:** | Susan Bassett |
| **IRB Committee:** | IRB-X |

**Date of Approval:** August 16, 2022

**Date of Expiration:** August 16, 2023

The JHM IRB approved the above-referenced Application.

JHM IRB is serving as the Single IRB for this study. **Please note that participating sites [other than Johns Hopkins] are not yet approved for human subjects research.**

**Adding Participating Sites [pSites]:** Requests for pSite approvals will need to be submitted via a pSite application before subject interaction may begin.

In reference to the study documents listed below, please note that template documents are not approved for use for enrollment.

**Approval includes:**

- Protocol – version 1 dated 7/25/2022
- One Written Informed Consent Form
- Three Oral Consent Scripts
- Five Recruitment Materials
- HIPAA Form 4
- 13 Supplemental Study Documents
- Risk Tiers Worksheet

To keep the JHM IRB application current we are assigning an Expiration Date as noted above. Prior to the expiration date, you will receive an email notification indicating that some action is required. If the Board has determined that a Continuing Review or Progress Report is required, you will need to submit Continuing Review or Progress Report prior to the expiration date. If the Board has determined that No Progress Report is required, you may run the administrative extend approval function.

IRB review included the following:

Clinical trials must be registered with a clinical trials registry that is electronically searchable and accessible to the public at no charge (i.e.: http://www.clinicaltrials.gov) as required by the September, 2007 FDA Amendments Act (affecting new and ongoing trials as of January 25, 2008). If this is a commercially sponsored trial, the Hopkins PI should consult with the commercial sponsor to assure that posting of the trial is in accord with terms of the study contract.

**45 CFR 46.116:** A waiver of consent was granted based on the following criteria: 1) the research involves no more than minimal risk to subjects; 2) the waiver will not adversely affect the rights and welfare of the subjects; 3) the research could not be practicably carried out without the waiver; and 4) the IRB will advise you if it is appropriate for participants to be provided with additional pertinent information after participation.

Use of an oral consent process.

**45 CFR 46.117(c)(1):** The only record linking the subject and the research is the consent document and the principal risk is loss of confidentiality. Each participant will be asked whether the participant wants documentation linking the participant with the research and the participant's wishes will govern.

The enrollment of the non-English speaking participants. If you are using a short form consent, follow the JHM IRB Guidance on Obtaining and Documenting Informed Consent of Subjects Who Do Not Speak English.

Your application included Johns Hopkins Community Physicians as a research site. You may not conduct this research at that site until you receive notification of site approval. If you have already received site approval, you may begin conducting research immediately.

**Progress Report Required:**

The Board determined that this research meets the criteria for submission of a Progress Report as an alternative to a Continuing Review Application. The Progress Report must be submitted using a Further Study Action and selecting progress report at least 6 weeks prior to the expiration date. Please note, the Progress Report **must** be submitted prior to the expiration date shown on this notice. If the Progress Report is not submitted prior to the expiration date all activity must stop. Before any research activity can resume, you must submit the progress report.

Research involving materials (data, documents, records, or specimens) that have been collected, or will be collected solely for nonresearch purposes (such as medical treatment or diagnosis).

Collection of data from voice, video, digital, or image recordings made for research purposes.

Research on individual or group characteristics or behavior (including, but not limited to, research on perception, cognition, motivation, identity, language, communication, cultural beliefs or practices, and social behavior) or research employing survey, interview, oral history, focus group, program evaluation, human factors evaluation, or quality assurance methodologies.

**Changes in Research:** All proposed changes to the research must be submitted using a Change in Research application. The changes must be approved by the JHM IRB prior to implementation, with the following exception: changes made to eliminate apparent immediate hazards to participants may be made immediately, and promptly reported to the JHM IRB.

**Unanticipated Problems:** All unanticipated problems must be submitted using a Protocol Event Report.

If this research has a commercial sponsor, the research may not start until the sponsor and JHU have signed a contract.

The JHMIRB is constituted to meet the requirements of the Privacy Rule at section 45 CFR 164.512(i)(1)(i)(B) and is authorized and qualified to serve as the Privacy Board for human

subjects research applications conducted by Hopkins' faculty members. The JHM IRB reviewed your request to waive or alter authorization for the above-referenced project. The IRB determined that all specific criteria for a waiver or alteration of authorization were met, as follows:
(A) The use or disclosure of protected health information involves no more than minimal risk to the privacy of individuals, based on, at least, the presence of the following elements;
(1) An adequate plan to protect the identifiers from improper use and disclosure;
(2) An adequate plan to destroy the identifiers at the earliest opportunity consistent with conduct of the research, unless there is a health or research justification for retaining the identifiers or such retention is otherwise required by law; and
(3) Adequate written assurances that the protected health information will not be reused or disclosed to any other person or entity, except as required by law, for authorized oversight of the research study, or for other research for which the use or disclosure of protected health information would be permitted;
(B) The research could not practicably be conducted without the waiver or alteration; and
(C) the research could not practicably be conducted without access to and use of the protected health information.

**Study documents:**

**Written Consent:**

Only consent forms with a valid approval stamp may be presented to participants. All consent forms signed by subjects enrolled in the study should be retained on file. The Office of Human Subjects Research conducts periodic compliance monitoring of protocol records, and consent documentation is part of such monitoring.
FINAL_Coleman_IRB00331706_CF_Providers_081622.docx

**Oral Consent Script:**
FINAL_Coleman_IRB00331706_ConsentScript_FGD_Staff_081622.docx
FINAL_Coleman_IRB00331706_ConsentScript_IDI_Staff_081622.docx
FINAL_Coleman_IRB00331706_ConsentScript_IDI_Patient_081622.docx

**Recruitment Materials:**
FINAL_Coleman_IRB00331706_TelephoneScreeningScript_081622.doc
FINAL_Coleman_IRB00331706_DearProviderEmail_081622.docx
FINAL_Coleman_IRB00331706_TelephoneScreeningScript_Provider_081622.doc
FINAL_Coleman_IRB00331706_MyChart_IDILetter_081622.docx
FINAL_Coleman_IRB00331706_ProviderFlyer_081622.docx

**HIPAA Form 4:**
FINAL_Coleman_IRB00331706_HIPAAForm4_081622.docx

**Additional Supplemental Study Documents:**
JHU-employee approval.pdf
sIRB Study Reliance Pre-Screen_CHIPPER.docx
UoM_data_elements.docx
APP A Gyn JHH history form.pdf
APPENDIX B.docx
JHHS-employee-approval
Appendix C GYN HIV Risk Tool-updated.docx
APPENDIX D -coordinating center.docx
Data collection sheet.docx
In-depth interview guide
Motivational Interviewing Training Presentation
Focus Group Discussion Guide
CCDA_DeIDprotocolApproval_IRB00331706.pdf

**Protocol:**
PrEP in Women Protocol-July_25-no male partner_jc_edits-clean.docx

**Johns Hopkins Study Team Members:**

Emmanuel Drabo, Marie Bielman, Runzhi Wang, Jamie Perin, Stephen Martin, Saumya Sao

The Johns Hopkins Institutions operate under multiple Federal-Wide Assurances: The Johns Hopkins University School of Medicine - FWA00005752, Johns Hopkins Health System and Johns Hopkins Hospital - FWA00006087
